# Virus particle propagation and infectivity along the respiratory tract and a case study for SARS-CoV-2

**DOI:** 10.1038/s41598-022-11816-2

**Published:** 2022-05-10

**Authors:** Dixon Vimalajeewa, Sasitharan Balasubramaniam, Donagh P. Berry, Gerald Barry

**Affiliations:** 1 Department of Statistics, Texas A & M University, College Station, TX USA; 2 Teagasc, Animal & Grassland Research and Innovation Center, Moorepark, Cork, Ireland; 3 School of Veterinary Medicine, University College Dublin, Dublin, Ireland; 4 School of Computing, University of Nebraska, Lincoln, USA

**Keywords:** Computational biology and bioinformatics, Mathematics and computing

## Abstract

Respiratory viruses including Respiratory Syncytial Virus, influenza virus and severe acute respiratory syndrome coronavirus 2 (SARS-CoV-2) cause serious and sometimes fatal disease in thousands of people annually. Understanding virus propagation dynamics within the respiratory system is critical because new insights will increase our understanding of virus pathogenesis and enable infection patterns to be more predictable in vivo, which will enhance our ability to target vaccine and drug delivery. This study presents a computational model of virus propagation within the respiratory tract network. The model includes the generation network branch structure of the respiratory tract, biophysical and infectivity properties of the virus, as well as air flow models that aid the circulation of the virus particles. As a proof of principle, the model was applied to SARS-CoV-2 by integrating data about its replication-cycle, as well as the density of Angiotensin Converting Enzyme expressing cells along the respiratory tract network. Using real-world physiological data associated with factors such as the respiratory rate, the immune response and virus load that is inhaled, the model can improve our understanding of the concentration and spatiotemporal dynamics of the virus. We collected experimental data from a number of studies and integrated them with the model in order to show in silico how the virus load propagates along the respiratory network branches.

## Introduction

Respiratory viruses are among the most transmissible viruses in the world and create a global health burden that contributes to thousands of illnesses and deaths annually. According to the American Center for Disease Control (CDC), influenza has killed between 12,000 and 61,000 people annually and caused approximately 460,000 hospitalizations per year in the United States. Respiratory Syncytial Virus (RSV) is estimated to cause close to 33 million cases of respiratory disease each year globally, with approximately 3 million hospitalizations^1^.

Once a respiratory virus enters the nose or mouth, it can move down the throat towards the lungs but much of the virus will deposit on the walls of the upper respiratory tract and then infect cells along the way, leading to amplification of the initial virus load. In this study, we are interested in virus spread from the upper to the lower respiratory tract. We thus consider that an infection has been controlled if it remained restricted to the upper respiratory tract. The dispersion of virus particles throughout the respiratory airway network system (i.e., the virus load along the respiratory tract) is influenced by multiple factors including respiratory pattern (i.e., inlet airflow rate), the concentration of virus particles in the inhaled air, also known as exposure level, the distance that the virus particles can travel through the respiratory airway network and importantly the strength of the immune response^2^. The bifurcation geometry of the airway network as well as the distribution of cell surface receptors that the virus can bind to also changes the virus dispersion along the respiratory network branches. For example, in the case of severe acute respiratory syndrome coronavirus 2 (SARS-CoV-2), the virus is known to use the Angiotensin Converting Enzyme (ACE2) receptor to bind to and enter cells^3^. Since cells expressing high ACE2 are not spread evenly throughout the respiratory system, some areas are more vulnerable to SARS-CoV-2 infection, thus creating a heterogeneous concentration distribution across the respiratory airway network system. Different variants can also have more or less affinity for ACE2 as well as a secondary receptor that SARS-CoV-2 uses, TMPRSS2^4^.

The temporal dynamics of the infection and the spreading pattern along the respiratory airway branches can be characterized and predicted effectively if the viral concentration dynamics (i.e., virus propagation, deposition, infection and reproduction along with immune response) can be characterized using modeling and simulations along with critical parameters that influence the system. From a personalization perspective, by being able to predict how the virus will impact a patient based on known criteria, medical staff will be able to make timely, informed and accurate decisions to prevent development of severe conditions. In the pharmaceutical industry, such model can benefit the development of drugs by allowing the in silico screening and characterization of inhibitory drugs prior to costly in vivo trials.

In the literature, we can find information about the temporal dynamics of virus infections over time, but not about the changes in viral dynamics along the respiratory airway branches. The main aim of this study was to develop a generic computational model for characterizing the dynamics of virus infection of the respiratory tract. The study uses SARS-CoV-2 as a case study to present how the derived model could be used to characterize the virus propagation dynamics in humans. The main contributions of this study are as follows:

1. Development of a temporal viral concentration model that can be used to analyze changes in virus dynamics as virus particles propagate along the branches of the airway in the respiratory system. This is achieved by accounting for the generation branching geometry of the respiratory airway network, and integrating models of the air flow that affects viral deposition and replication, and its impact on the virus spread distribution along the airway generations.
2. Modelling the spatial and temporal dynamics of the virus particle within the respiratory tract, by considering parameters such as the breathing rate and dynamic viral pleomorphic size change with respect to the changes from the inhaled dosage.
3. Sensitivity analysis of the viral propagation along the airway generation branches considering factors such as the exposure level, cellular receptor availability and immune response and then characterizing the temporal dynamics in virus load with respect to different patient parameters such as age, gender, and lifestyles.

## Methods

**Figure 1:**
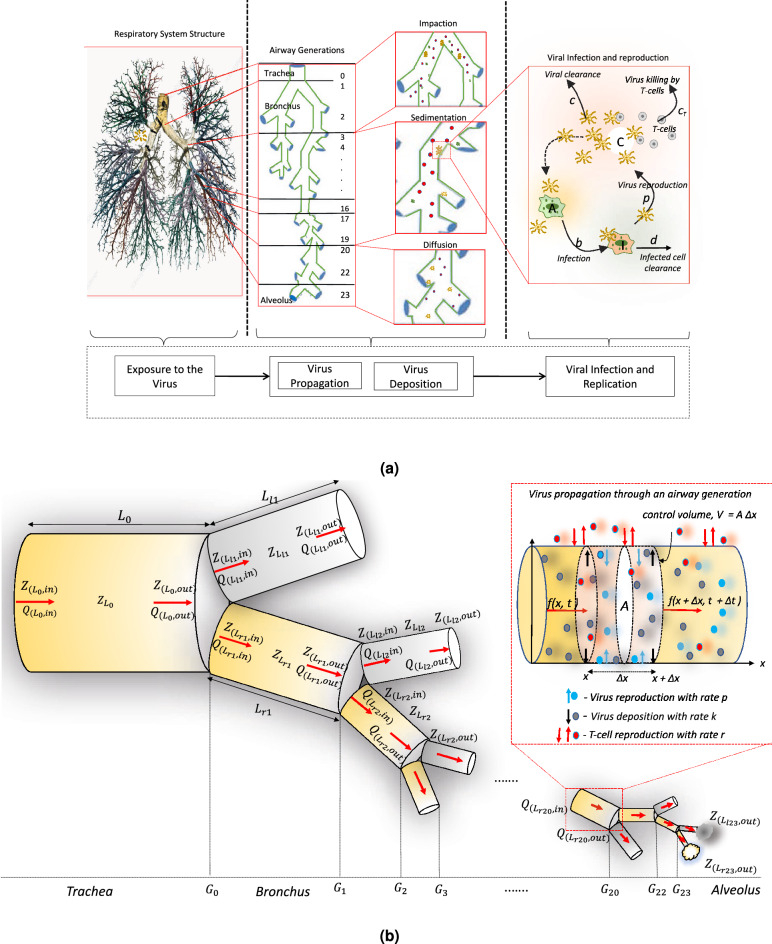
An overview of the computational modeling approach; (**a**) computational model for exploring the temporal viral propagation dynamics down the branch generation of the respiratory tract considering the virus propagation, deposition, and infection dynamics along the airway tract and (**b**) bifurcation of the respiratory (left - *l* branch and right - *r* branch) airways. The subscript notation *in* and *out* are used to denote the inlet and outlet pressure (*P*), airflow rate (*Q*) and impedance (*Z*) in each airway and virus particle propagation through a control volume of an airway with deposition rate *k* and reproduction rate *p* (this image was created by using Microsoft PowerPoint version 16.57 and can be accessed here).

The main steps in the process of deriving the computational model for the virus propagation dynamics along the respiratory airway generations are illustrated in Fig. 1a. The airway generations are formed by subdivision of the larger airways in the respiratory tract into smaller airways (as in tree structure) and there are 23 airway generations. Fig. 1b depicts the respiratory airway generation structure that is used in this study to characterize the viral particle concentration and its distribution along the respiratory tract. There are three main elements to the model which include the airflow dynamics, virus concentration dynamics based on particle propagation and deposition, and the virus infection dynamics (complete derivation of each of these processes are provided in the Supplementary Information (SI)).

### Respiratory air flow dynamics

To determine airflow dynamics, the respiratory generation model in this study is taken from literature^5^. When virus particles enter through the mouth or nose, they flow through the respiratory tract. To determine the amount of virus particles that enters a particular airway generation, the air flow velocity *u* through that branch is required. To compute airflow velocity in each airway generation, this study takes into account the bifurcation transformation between the large airways that separates into smaller branches with reduced lengths and diameters as illustrated in Fig. 1a.

Suppose an airway of length $L_0$ branches into left and right branches of length $L_{l1}$ and $L_{r1}$ as illustrated in Fig. 1b. Based on the conservation of air flow, at the first bifurcation (i.e., $G_0$), the conditions $Q_{L_0,out} = Q_{L_{l1},in} + Q_{L_{r1}, in}$ and $P_{L_0,out} = P_{L_{l1},in} = P_{L_{r1}, in}$ hold at the bifurcation junction; where *Q* is the flow rate, *P* is the pressure, $L_{l1}$ and $L_{r1}$ are respectively the lengths of the left (*l*) and right (*r*) branches, and the numbers along with *l* and *r* refers to the airway generation number. In addition, the subscript *in* and *out* stands respectively for the inlet and outlet of the branch. When air flows down the airway, there is a drop in pressure as well as resistance against the air flow (termed as impedance). We adapt these properties and model the flow using transmission line circuit theory, where its equivalence is voltage drop in response to changes in the current flow^6^. Hence, following the concepts from transmission line circuit theory, the impedance (*Z*) at the outlet of the parent airway ($Z_{L_0, out}$) is related to the impedance at the inlet of the branching airways as $\frac{1}{Z_{L_0,out}} = \frac{1}{Z_{L_{l1}, in}} + \frac{1}{Z_{L_{r1}, in}}$. This results in the impedance at the inlet being expressed as $Z_{L_0, in} = Z_{L_0} + Z_{L_0,out}$, where $Z_{L_0}$ is the impedance of the airway (see Eq. 5 in SI for the derivation of $Z_{L_0}$). Based on this, the inlet air flow rates to the branches and the outlet pressure of the parent airway can be represented as

1 $$\begin{aligned} Q_{L_{l1},in}=\, & {} Q_{L_0,out}\frac{Z_{L_{r1},in}}{Z_{L_{l1},in}+Z_{L_{r1},in}}, \quad \text {and} \quad Q_{L_{r1},in} = Q_{L_0,out}\frac{Z_{L_{l1},in}}{Z_{L_{l1},in}+Z_{L_{r1},in}} , \end{aligned}$$

2 $$\begin{aligned} P_{L_0,out}= \,& {} Q_{L_0,out} Z_{L_0,out}, \quad Q_{L_{l1}, in} = Q_{L_{l1},out} \quad \text {and} \quad Q_{L_{r1}, in} = Q_{L_{r1},out} . \end{aligned}$$

To compute the airflow rates, as well as the velocity profiles over the respiratory tract, we use a backward calculation procedures, which starts with the impedance at the outlet of the last airway (i.e., 23rd generation’s $Z_{(L_{l23}, out)}$ and $Z_{(L_{r23}, out)}$ as represented in Fig. 1b). As an example, the procedure for the 23rd and 22nd generation ($G_{22}$ and $G_{23}$) airflow rate is as follows:

1. The impedance of the $G_{23}$, $Z_{23}$, as well as the impedance at the inlet of this airway, $Z_{(23, in)}$, is computed (see Eq. 1–9 in SI for formulas).
2. The impedance at the outlet of the $G_{22}$, $Z_{(22, out)}$ is then computed based on the impedance values of step 1 ($G_{23}$).
3. Step 1 is repeated to compute the impedance of the $G_{22}$, $Z_{22}$, as well as the impedance at the inlet, $Z_{(22, in)}$.

These steps are repeated up to the initial airway generation ( i.e., $Z_{0}$ as represented in Fig. 1b) to compute the impedance of each airway generations. Then, the flow rate *Q* into each airway generation is computed using Eqs. (1) and (2). Having derived the flow rate into the airway, the air flow velocity *u* is then computed using the expression $Q = Au$, where $A = \pi (d_L/2)^2$ is the cross sectional area of the airway of diameter $d_L$. The real physiological parameters, length (*L*) and diameter of the airway generations ($d_L$) given in Table 1 in the SI are used for computing the air flow velocity of each airway generation.

The air enters into the respiratory tract under external forces that includes both turbulence as well as gravity, and the impact of these forces gradually decreases once we go deeper into the lung. Hence, the propagation of virus particles contained in the airflow (i.e., concentration) can be determined using the advection-diffusion property^7,8^.

### Virus concentration dynamics

The advection mechanism is the dominant force that facilitates the propagation of particles and their deposition along the upper airways due to the high airflow velocity, which in turn, causes turbulence in the airflow. This differs from the lower branches within the respiratory track, which is dominated largely by pure diffusion. These factors are next taken into account to compute virus concentration in each airway generation.

The concentration of the viral particle in the *i*th airway generation, $C_{G_{i}}$, will depend on the quantity of virus that is in the previous airway generation (i.e., $C_{G_{i-1}}$). This means that the virus infection level (or the virus concentration) of the *i*th airway generation ($G_i$) depends on the virus infection level of the $(i-1)$th airway generation ($G_{i-1}$) and its propagation along the branch. Moreover, the virus particle deposition rate and the virus replication rate in the host cells along with the respiratory system geometry such as the airway length and diameter also contributes to the virus infection level. However, virus flow is unlikely to be completely unidirectional, but a recent study on influenza virus indicates that infection initiates in the upper respiratory tract and then spreads predominantly from the upper respiratory tract implying that the amount of virus in the lower respiratory tract that moves to the upper respiratory tract and out is low^9^. With this factors in mind and with the acknowledgment that a small proportion of virus may move upwards, we have focused on unidirectional movement in our model for now but this can be easily adapted as more experimental data becomes available.

By taking these factors into account, this section presents how the advection-diffusion mechanism of the virus particle propagation can be utilized to compute the concentration over an airway generation $G_i$. When the virus particles travel down the respiratory tract under the advection-diffusion mechanisms, they deposit on the airway walls and bind onto ACE2-expressing cells to initiate infection and subsequently the replication process. Considering the viral infection and replication process depicted in Fig. 1b and the mass balance of the virus particles in the control volume, the change in the virus concentration $G_i$ is represented as

3 $$\begin{aligned} \frac{{\partial C_{G_i}(x,t)}}{{\partial t}} + u_i\frac{{\partial C_{G_i}(x,t)}}{{\partial x}} - D \frac{{\partial C_{G_i}(x,t)^2}}{{\partial x^2}} + (p-k)C_{G_i}(x,t)= 0, \end{aligned}$$

where the generation number $i= 1,2, \ldots , 23$, *k* and *p* are respectively the virus deposition and reproduction rates, *D* is the air diffusion coefficient, $u_i$ is the velocity of virus particles in the *i*th airway generation and *x* stands for the direction of the virus propagation (i.e., downward of the respiratory tract). Given the initial inlet virus concentration that a person consumes through the mouth or nose is $C_{0}$ (i.e., $C_0 = C_{G_0}(x =0,t=0) = C_{G_0}$), the solution of Eq. (3) can be derived as

4 $$\begin{aligned} C_{G_i}(x,t) = \frac{C_{G_{i-1}}}{2\sqrt{4\pi D t}}exp{\left( -\frac{1}{4} \frac{(x-u_it)^2}{Dt} - (k-p)t\right) }, \end{aligned}$$

where $C_{G_{i-1}} = 2C_{G_0}$ only at $t=0$ as $C_{G_{i-1}}$ divides equally into two when the virus enters the branches of the $G_{i}$ generation for $i =1, 2, 3, \ldots , 23$.

In order to simplify virus concentration dynamics expression given in Eq. (4) by removing unknown terms, we consider the steady state of the airflow dynamics. At steady state, $\frac{\partial {C_{G_i}}}{\partial {t}} = 0$ and $u_i$ is also a constant. This means that Eq. (3) can be simplified into $Da^2 -u_ia - (k-p)= 0$ with $a = \frac{\partial {C_{G_i}}}{\partial {x}}$. This is a quadratic expression and thus, the solution is $a = \frac{u_i \pm \sqrt{u_i^2 + 4D (k-p)}}{2D}$. Since concentration $C_{G_i}$ declines over time and $C \ge 0$, $a = \frac{u_i - \sqrt{u_i^2 + 4D (k-p)}}{2D}$. Using this value of *a*, the virus concentration at the steady state is expressed in Eq. (5) (see SI for more details). The change in the virus concentration along an airway that includes the effects of virus reproduction and their deposition on the airway walls is represented as

5 $$\begin{aligned} C_{G_i}(x) = \frac{u_iC_{G_{i-1}}}{u_i + \sqrt{u_i^2 + 4D(k-p)}} exp{\left( \frac{\left[ u_i- \sqrt{u_i^2 + 4D (k-p)}\right] x}{2D} \right) }, \end{aligned}$$

for $$i = 1, 2, \ldots , 23$$.

Next subsection describes the methods for computing virus deposition rate (*k*) and followed by the virus reproduction rate (*p*), which are required for determining the virus concentration using Eq. (5).

#### Particle deposition rate

The virus deposition (*k*) mainly takes place under three main mechanisms, impaction ($k_I$), sedimentation ($k_S$) and diffusion ($k_D$). Hence, the final particle deposition rate *k* is computed as the sum of all deposition rates and is represented as

6 $$\begin{aligned} k = k_{I} + k_{S} + k_{D}. \end{aligned}$$

This study uses the formulas given in the study^5^ to compute $k_I$, $k_S$ and $k_D$ and more information about them can be seen in^5,10,11^.

1. *Impaction (*$k_I$*):* Virus deposition due to impaction takes place in response to virus particles which cannot flow evenly along their trajectory due to their inertia and surroundings. These barriers increases with particle size and flow rate and mostly occurs in the upper airways due to high velocity. In our study, $k_I$ is computed as $k_I = \frac{1.3}{L} \frac{\rho _p d_p^2u}{18\mu d_L}$, where *L* is the airway length, $\rho _p$ is the virus particle density, $d_p$ is the virus particle diameter, $\mu$ is the air viscosity and $d_L$ is the airway diameter.
2. *Sedimentation (*$k_S$*):* Sedimentation occurs when the virus particles move under the gravitational force, and they settle on the inner surface of the airways in the upper respiratory region, and is computed as $k_S = \frac{2Q}{\pi L}( 2 B\sqrt{1 - B^{2/3}}- B^{1/3}\sqrt{1-B^{2/3} } + sin^{-1}(B^{1/3}))$^5^, where $B = \frac{3 \pi L u_g}{16ud_L}$ and $u_g$ is the settling velocity of the virus particles and is known as the maximum velocity that a virus particle can achieve (see Eqs. 32–36 in SI for more information about deriving $u_g$).
3. *Diffusion (*$k_D$*):* Diffusion-based particle deposition acts on the virus particle resulting from Brownian motion. This increases in response to decreasing particle size $d_p$ and and flow rate $Q_0$, and is represented as $k_d = 1-0.819e^{-14.63B} - 0.0976 e^{-89.22 B} - 0.0325e^{-228B} - 0.0509e^{-125.9B^{2/3}}$, where $B = \frac{\pi D L}{4Q}$, *D* is the diffusion coefficient, *Q* is the flow rate, and *L* is the airway length.

### Virus infection dynamics

Virus concentration will vary across the respiratory tract as infection progresses (Fig. 1a). The SARS-CoV-2 uses ACE2 as its main cellular receptor, enabling entry into a number of different cell types found in the respiratory tract (e.g., tracheal and bronchial epithelial cells, type 2 pneumocytes, macrophages)^12^. ACE2 expression in the respiratory system is not stable but evidence is not clear yet as to how that expression precisely changes and where in the system those changes occur. For this reason, we assume that expression does not change as a result of infection. Going forward, as more data becomes available, this can be easily incorporated into the model. In contrast, the immune response to infection has been well characterized, although data about the immediate early immune response during the acute phase of the infection is relatively limited. The immune response includes different components such as interferons (IFNs), cytotocxic T-cells (CTL) and antibodies (Abs), and has two main phases: the innate and the adaptive immune response^13^. It is of course challenging to measure the immediate response to a SARS-CoV-2 infection but correlative studies indicate that IFNs play an important role in protecting against severe disease. Impaired IFN response appears to correlate with worse outcomes^14^ while genome wide studies have shown particular genotypes in key IFN pathway related genes correlate with more severe outcomes^15,16^. Alongside this, the limited T-cell response, an early responder to infection in its own right, is also associated with more severe disease suggesting that a more robust response may help to control the infection before it gets out of control. Because of the complexities of the immune response, the variations within it and the lack of absolute clarity, it is challenging to account for each pathway in the overall response, but this is a complexity that can be added in layers as the process becomes more refined. For now, we will focus on the T-cell response as the *in vivo* data is clearer.

The model (Eqs. 7–10) represents the relationship between viral particles (*C*) and immune response dynamics by assuming T-cell (*T*) proliferation by viral infection follows a Michaelis Mention growth and the relationship which explains the activation of T-cell proliferation by virus infection is log-sigmoidal form, along with the susceptible cell (*A*) infection and infected cell (*I*) generation. However, this model has originally been developed for characterizing the dynamics in influenza virus and the width of the log-sigmoidal function was considered as 1 (i.e., $m =1$). So, to make this model more specific to SARS-CoV-2, a modification was applied to the *m* value and it was selected as 2. That is because the study^3^ has justified that the best fit value for *m* is 2 for explaining SARS-CoV-2 virus dynamics based on clinical data collected from COVID-19 patients.

7 $$\begin{aligned} \frac{\partial {A}}{\partial {t}}= & {} - b A C, \end{aligned}$$

8 $$\begin{aligned} \frac{\partial {I}}{\partial {t}}= & {} b A C - dI, \end{aligned}$$

9 $$\begin{aligned} \frac{\partial {C}}{\partial {t}}= & {} pC\left( 1 - \frac{C}{K}\right) - cC - c_T C T,\end{aligned}$$

10 $$\begin{aligned} \frac{\partial {T}}{\partial {t}}= & {} s_T + r T \left( \frac{C^m}{C^m + k_T^m} \right) - d_T T, \end{aligned}$$

where, susceptible cells *A* infection rate is *b* and infected cells *I* removing rate is *d*. The virus particles, *C*, are produced at a rate *p* whilst they are cleared at a rate *c* and inactivated by the immune response at a rate $c_T$. The infected cell killing rate by the immune response is given as $c_TCT$. *K* is maximum carrying capacity (or maximum virus load) and $T_0 (= s_T/d_T)$ is T-cell concentration before infection (initial immune response); $d_T$ is the half life of T-cells and $s_T$ represents T-cell homeostasis, $k_T$ is half saturation constant of T-cells, and *r* is the T-cell proliferation rate (see^3^ for more information about this model).

## Results

The computational outcomes presented below are generated by using the parameter values listed in Table 1, unless otherwise mentioned for specific analysis (Python software was used for simulations and main python codes used for simulations are available here).

**Table 1:** Parameters and notations along with their values used in simulations.

Symbol	Parameter	Value
**Airflow characteristics**		
$\rho _f$	Air density	1.2 $\times 10^{-4}$ (gcm$^{-3}$)
$\mu$	Air viscosity	1.81 $\times 10^{-4}$ (g cm^1^s$^{-1}$)
$Q_0$	Inlet airflow^5^	30 (lmin$^{-1}$)
*g*	Acceleration of gravity	980 (cm s$^{-2}$)
$\lambda$	Mean free path of gas molecules^17^	0.066 $\times 10^{-4}$ (cm)
$K_B$	Boltzmann constant	1.38$\times 10^{-23}$ (JK$^{-1}$)
*T*	Room Temperature	298 (K)
*L* and $d_L$	Airway Length and diameter^5^	Table 1 in SI
**Virus characteristics and data**		
$C_0$	Inlet virus concentration^3^	$1\times 10^{7}$ (Copies /ml)
*A*	ACE2 concentration^12^	${\mathcal {N}}(5.83, 0.71)$(Copies/ml)
$d_p$	Diameter of virus particle^18^	60–140 (nm)
$\rho _p$	Density of virus particle^19,20^	1.18 (gcm$^{-3}$)
*p*	Virus reproduction rate^3^	8.2 (day$^{-1}$)
*c*	Virus clearing rate^3^	0.6 (day$^{-1}$)
*b*	Virus infection rate with susceptible cells^3^	$3.9 \times 10^{-7}({\rm Copies}/{\rm ml})^{-1} {\rm day}^{-1}$
*d*	Infected cell clearing rate^3^	$4.71 ({\rm day}^{-1}$)
**Immune response data**		
*K*	Maximum carrying capacity^3^	$10^8$ (Copies/ml)
$c_T$	Virus killing rate by immune T-cells^3^	$5.01 \times 10^{-8}$ (${\rm day}^{-1}$)
*r*	Immune T-cell proliferation rate^3^	5.89 (${\rm day}^{-1}$)
$d_T$	Half life of T-cell^3^	$2.9 \times 10^{-2}$ (${\rm Copies}/\mu {\rm l}$)
$k_T$	Half saturation constant^3^	$7.94 \times 10^7$ (Copies/ml)
$T_0$	T-cell level absence of viral infection^21^	$955 - 2860$ (${\rm Copies}/\mu {\rm l}$)
$T_0$ for age^22^	–	$2907^{a}, 2533^{b}$, $1361^{c}$ (${\rm Copies}/\mu {\rm l}$)
$T_0$ for gender^22^	–	$2841^{m}, 3226^{f}$ ($Copies/\mu l$)
$T_0$ for lifestyles^2^	–	$1690^s$, $1551^{ns}$,
		$1550^{al}$, and $1595^{nal}$ (${\rm Copies}/\mu {\rm l}$)

*m*—male, *f*—female, *a*—20–40, *b*—40–60, *c*—60–80 ( years), *s*—smoking, *al*—alcohol, *ns*—not smoking, and *nal*—non-alcoholic.

### Virus propagation through the airway generations

**Figure 2:**
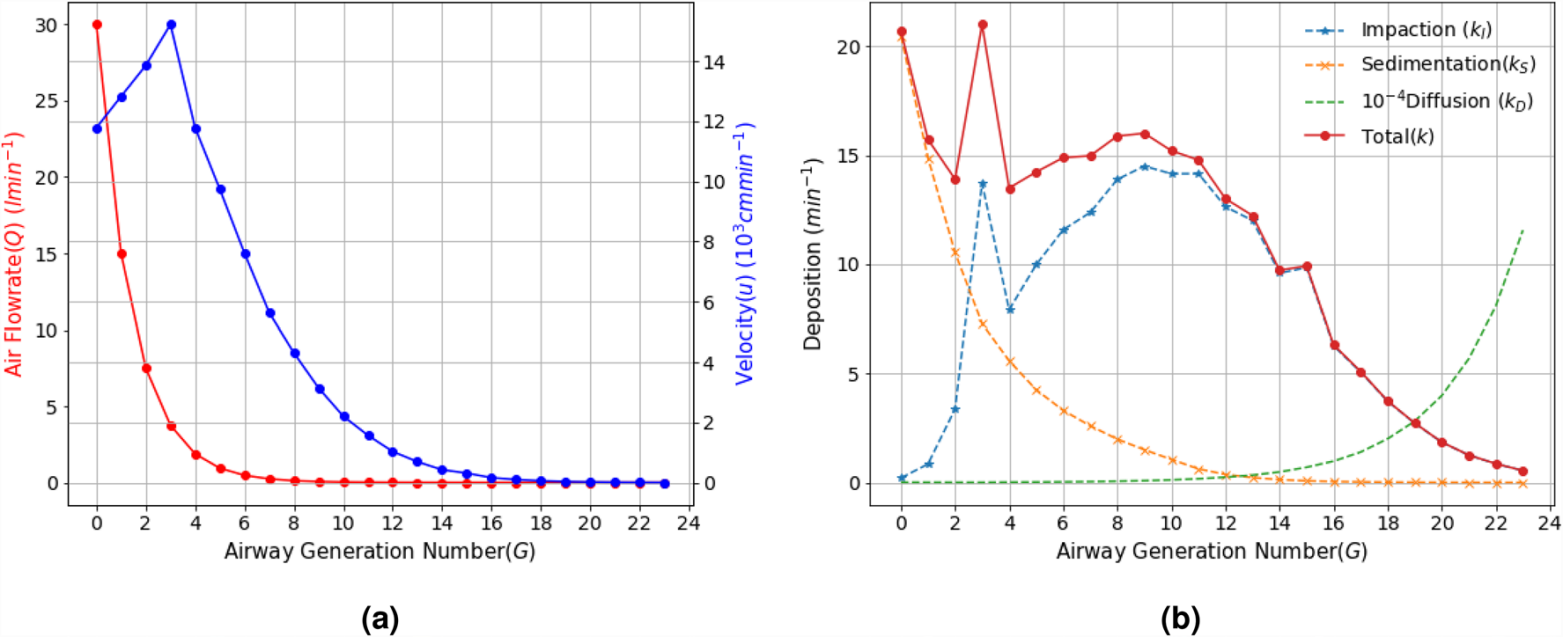
Airflow rate, velocity and virus deposition. The change in airflow rate *Q* and velocity *u* (**a**) along with deposition *k* (**b**) over the airway generations in the respiratory system when the inlet airflow rate $Q_0 = 30$lmin^−1^.

The term airway generations is used to describe what part of the respiratory tract the virus is in. The higher the generation the deeper into the respiratory system it is. Generation numbers go from 0 (top of trachea/mouth/nose) to 23 (bottom of airway/alveoli). To model how a virus particle moves through the respiratory system, based solely on air movement, we used the derived model in Eq. 1 to compute the airflow rate *Q* and the velocity *u* in each airway generation by using real physiological parameters such as the length and diameter of the airway generations^5^. Dimensions will change in younger children and this can be added to the model if focusing on children^23^. Figure 2a shows both the change in *Q* and *u* over the 23 generations when air enters through the nose or mouth at a rate of 30 lmin$^{-1}$. The airflow rate reduces to nearly half its value as it progresses deeper into the respiratory system due to the bifurcation of larger airways that are separated into smaller branches. The velocity shown in Fig. 2a also reduces with higher airway generations, showing that the dominant viral propagation is governed by the advection and diffusion process in the upper and lower parts of the respiratory system. Figure 2b depicts virus deposition under sedimentation and impaction, and shows again that this is higher in the upper airways, while the particle deposition due to diffusion increases with increasing airway generation number.

**Figure 3:**
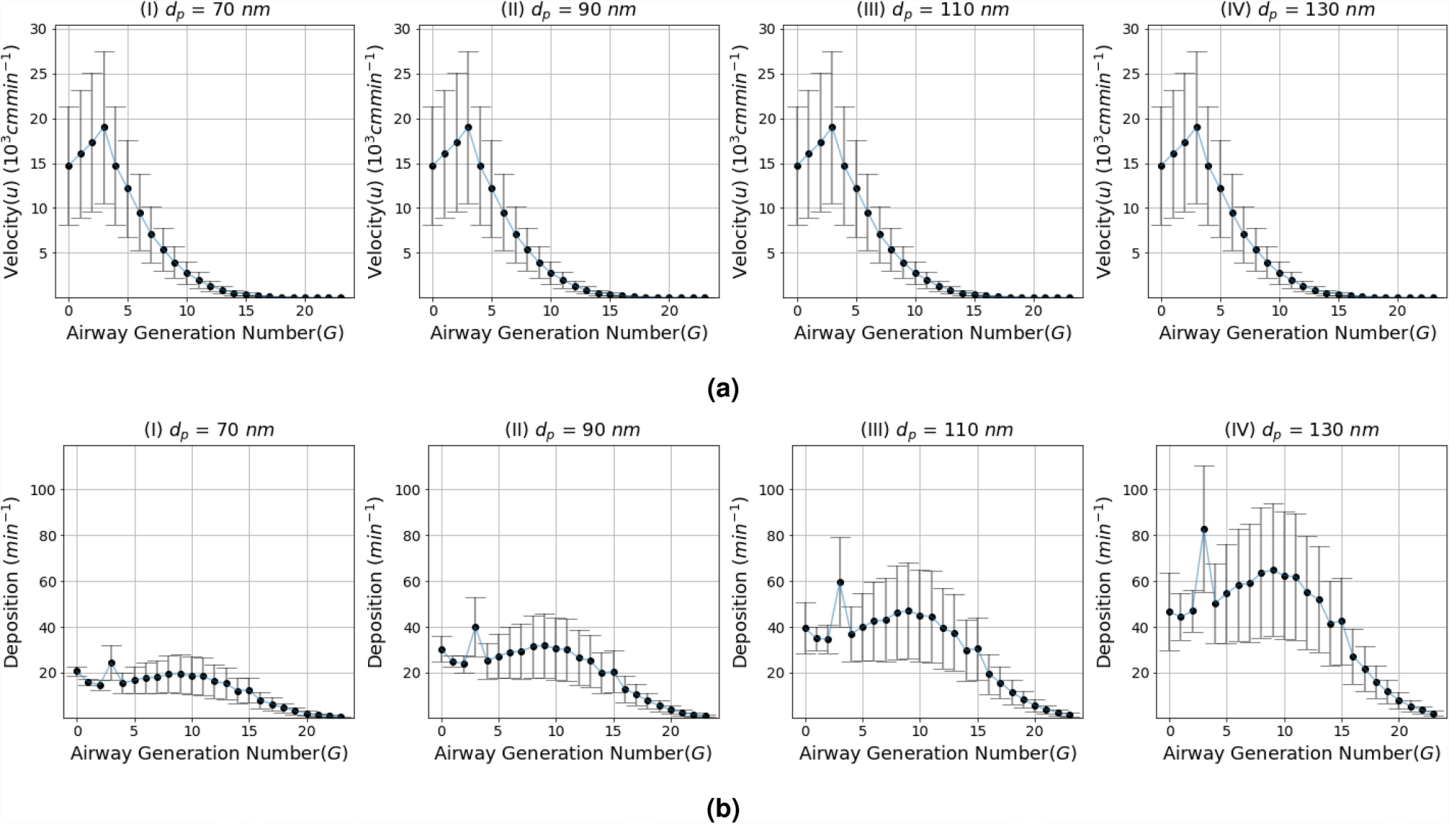
The impact of the virus particle diameter $d_p$ on the airflow velocity *V* and particle deposition rate *k*; (**a**) airflow velocity *V* and (**b**) deposition rate *k* with respect to variations in the diameter of the virus particle $d_p$ in the 70–130 $\mu m$, ($Q_0$ varies in the rage 15–60 lmin^−1^).

The virus shape is pleomorphic (i.e., changes shape) but is predominantly elliptical or spherical. Also, as larger particles are mostly trapped in the upper airways, while smaller (e.g., micrometer scale) particles propagate deep down the lungs. Therefore, we also analyzed the impact of the virus particle shape on its propagation through the respiratory system based on changes in its diameter $d_p$ between 60 and 140 nm^18^. In terms of velocity and deposition, Fig. 3a$(I-IV)$ and Fig. 3b$(I-IV)$ depicts the impact of change in the virus diameter with inlet airflow rate on the velocity and deposition along the respiratory system. The analysis includes the average velocity and deposition with the error bars computed for several $Q_0$ values for the range $Q_0 = 15, 30, 45, 60$ lmin^−1^. It can be observed that the temporal dynamics in the virus deposition rate over the first 15 airway generations is considerably higher. In addition, the impact of increasing inlet airflow rate $Q_0 (= 15 - 60 )$ lmin^−1^ presented in Fig. 4 in the SI shows that the deposition in the upper respiratory tract also increases.

### Virus concentration dynamics along the airways

To understand the virus concentration dynamics as it flows along the airways, our analysis assumes a virus concentration of $10^7$ Copies/ml (i.e., $C_0$)^24^ (or *Infectiousparticles*/*ml*) entering the respiratory tract (i.e., $C_0$) at a breathing airflow rate $Q_0 = 30$ lmin^−1^. We also take into account that the density of ACE2 expressing cells such as alveolar type-II and epithelial cells is greater around the mouth, nose and alveolar compared to other parts of the respiratory tract^7^. This is achieved by assigning ACE2 concentration values for each airway generation based on the Gaussian distribution, where we averaged higher ACE2 expression ($\sim 6-7$ Copies/ml) around the trachea and alveolar (i.e., $\sim 1-2$ and $\sim 21-23$ airway generations) compared to the other airway generations ($\sim 4-5$ *Copies*/*ml*), but with the same variance.

**Figure 4:**
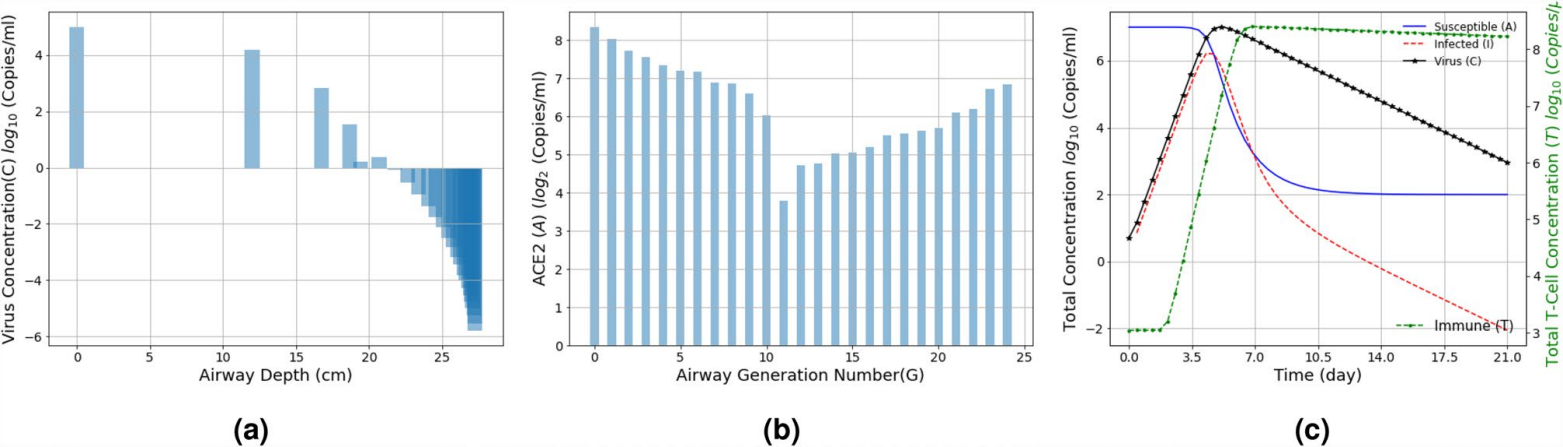
Virus deposition along the airway generation branches and infection (**a**) its changes with respect to the initial virus load and (**b**) distribution of ACE2 expressed cells over the respiratory airways, and (**c**) simulation of total viral particles (*C*), infected cells (*I*), ACE2 (*A*) and immune T-cell (*T*) concentrations over time as a result of the viral infection; T-cell count in the absence of virus $T_0 = 10^3 {\rm Copies}/\mu {\rm l}$(a value within the $T_0$ range given in Table 1).

**Figure 5:**
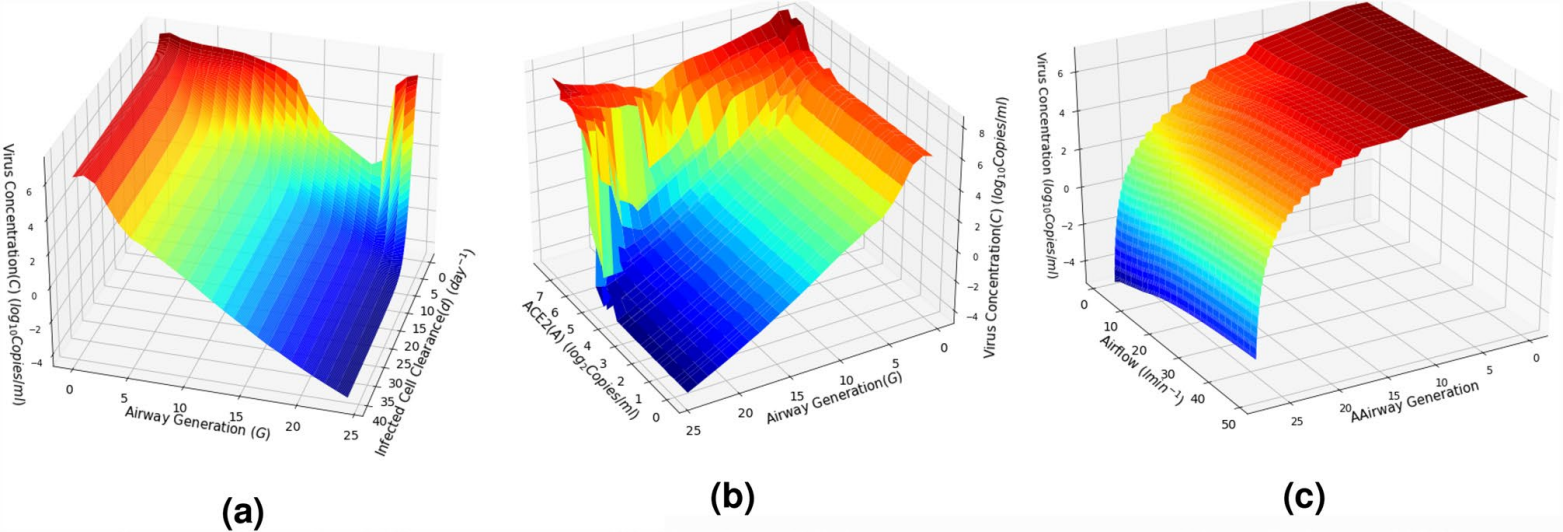
Virus load over the respiratory airways for 21 days with respect to the changes in three different parameters; **(a)** Infected cell clearance rate *d* ($Q_0= 30$ lmin^−1^), **(b)** density of ACE2 expressed cells ($Q_0= 30$ lmin^−1^ and $d = 4.71$ ${\rm day}^{-1}$), and **(c)** inlet airflow rate $Q_0$ ($d = 4.71$ ${\rm day}^{-1}$); where the virus concentration is computed while varying the virus particle diameter $d_p$ over the range 70–140 $\mu m$ and fixed initial virus concentration $C_0 = 10^7$ Copies/ml.

Figure 4a shows the change in the virus concentration using Eq. (5), while Fig. 4b depicts the change in ACE2 expression levels over the airway generations. It can be observed that the abundance of virus is higher in the upper airways (e.g., trachea) compared to the high generations that are deep in the lungs. This aligns with the clinical observation that the possibility of initial infection is likely around the mouth, nose and throat^3^. Figure 4c presents a simulated end-to-end concentration evolution of the virus over three weeks to illustrate the effects of the infection and replication process along with the T-cell response once the virus has entered the airway, which is based on Eq. (7). Based on the COVID-19 clinical data and virus characteristics listed in Table 1, Fig. 4c shows the general viral infection dynamics (not specific to any airway generation) modelled in Eqs. (7)–(10). Virus concentration reaches its peak ($\approx 10^7$ Copies/ml) 5–7 days after the initial infection and reduces as the immune system responds against the virus. This time period may shorten if prior immunity is present. This prediction is in line with clinical observations investigated in a previous study^25^, which justifies the need for self-isolation between 7 and 14 days post-infection as the virus concentration would be at its highest during this period, which increases the probability of onward transmission.

Virus concentration is impacted by apoptosis driven by infection (Fig. 5a), ACE2 expression distribution (Fig. 5b), and airflow rate (Fig. 5c). Many respiratory viruses with cause cell to die by apoptosis. Cytotoxic T-cells will also trigger cellular apoptosis if cells are infected. Figure 5a shows that the virus load decreases in the respiratory tract as the number of infected cells die and this was particularly prominent in the lower generation levels, where early infection occurs. Virus concentration decreased through generations until the highest generations where virus concentrations spiked to a high level. Fig. 5b illustrates that the model predicts that when ACE2 expression is high, virus concentration will increase, identifying that virus affinity to ACE2 is a major factor in where the virus will predominantly replicate. Interestingly, when airflow is increased, our model predicts that it does not increase the likelihood of virus concentrations increasing in the lower respiratory tract (Fig. 5c).

**Figure 6:**
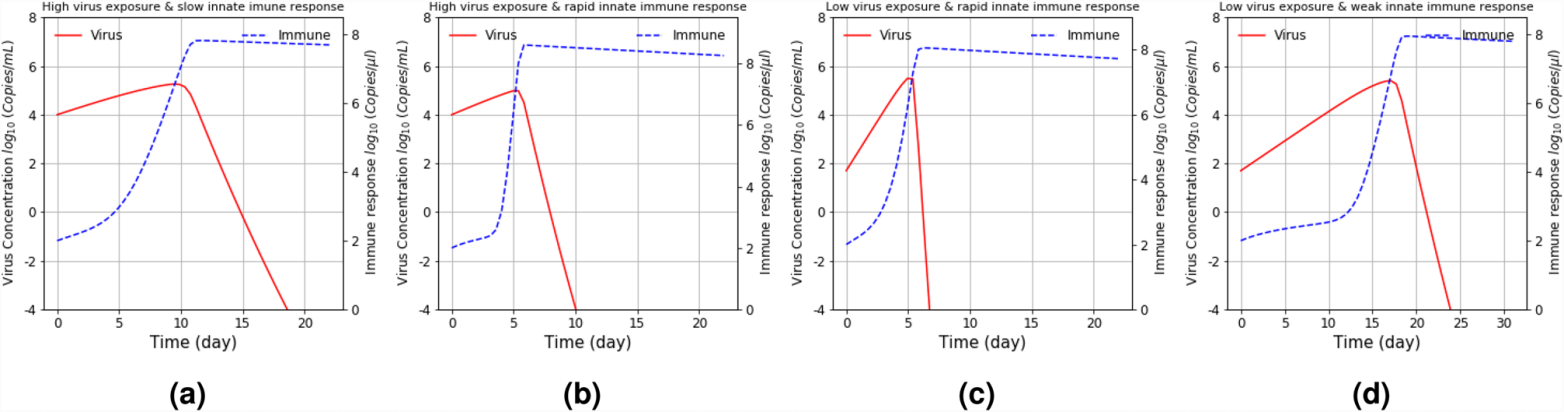
Impact of the immune response and the exposure level on the overall virus load, given that the initial T-cell level $T_0$ is $10^3$ ${\rm Copies}/ \mu {\rm l}$ and the infected cell clearance rate $d = 4.71 (day^{-1})$; (**a**) high exposure level ($C_0 = 10^4$ Copies/ml) and slow immune response ($r = .5$ ${\rm day}^{-1}$) and (**b**) high exposure level ($C_0 = 10^4$ Copies/ml) and rapid initial immune response ($r = 10$ ${\rm day}^{-1}$), (**c**) low exposure level ($C_0 = 50$ Copies/ml) and rapid initial immune response ($r = 10$ ${\rm day}^{-1}$), and (**d**) low exposure level ($C_0 = 50$ Copies/ml) and slow immune response ($r = .5$ ${\rm day}^{-1}$).

Naturally, to identify major factors impacting virus distribution through the respiratory system it is important to remember that each factor does not act in isolation, however, our model is able to isolate each factor and project it’s influence on virus dynamics. The immune response clearly plays a role in this. As illustrated earlier, cell death is important but the cellular and antibody based immune responses are vital too. Although an antibody response may take a few days to optimally respond to an infection, the early cellular response is the front line of defense and plays a major role in limiting virus access to the lungs. Figure 6a–d depicts how the virus concentration dynamics changes when there is variations in the speed of the immune response (*r* is 0.5–10 ${\rm day}^{-1}$) and exposure dose $C_0$ (50-$10^4$ Copies/ml). In this case, the maximum virus load carrying capacity *K* is $10^6$ Copies/ml, while the virus reproduction and clearance as well as the infected cell clearance rates were fixed based on values in Table 1. Initially the virus concentration increases until it reaches a peak and eventually decreases. In response to the increase in virus concentration, the immune response also increases. (i.e., immune response by T-cells increases, both in number and efficacy). However, a high peak virus persistence is observed over a long period when the T-cell immune response is slow or at a low level compared to the initial virus exposure level. Also, when the strength of the early immune response is greater than the virus exposure level in the model, the virus concentration rapidly declines after reaching its peak.

### Temporal dynamics in virus load over the respiratory system

**Figure 7:**
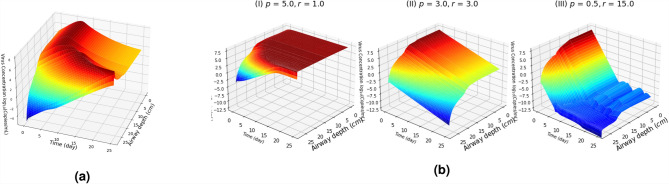
Virus load in the respiratory tract: **(a)** temporal dynamics in the virus load in the respiratory airway generations over time with the immune response by T-cells and **(b)** impact of the virus production rate *p* and the immune response by T-cells *r* on the temporal dynamics in virus load for three cases: $p>r$, $p=r$, and $p<<r$ (other parameters are fixed to the values given in Table 1).

To expand the previous results further, it is important to consider virus concentration throughout the respiratory system rather than just overall values. Figure 7a shows the change in virus concentration over the respiratory tract when $p = 8.2 {\rm day}^{-1}$ and $r = 5.89 {\rm day}^{-1}$. The virus concentration is high in the upper and become higher in the lower respiratory tract over time. Figure 7b shows the impact of the virus reproduction rate *p* and the immune response by T-cells *r* on the change in virus concentration over the respiratory tract for three cases: $p>r$, $p=r$, and $p<<r$. As observed in these graphs, the time to reach the peak virus load increases with the airway generations. The virus will continuously flow downwards into the lower parts of the respiratory tract, thus amplifying any replication that is taking place in the lung. Then, the virus load decreases after reaching the peak as susceptible cell count reduces and the immune response by T-cells increases. As seen in Fig. 7b, the time taken to reach the peak and then decrease is much longer when $p\le r$ compared to that of when $r>>p$. That is, high virus concentration persists when the virus production rate *p* is much larger compared to the immune response by T-cells and is more likely to reach high concentrations in the lung. The model also illustrates that the concentration in the lung can increase initially but an efficient immune response is vital to rapid reduction of virus, both in the lung and upper respiratory tract. Moreover, a rapid immune response clears virus by 10 days approximately as shown in Figs. 4b,c and 6.

In addition, Fig. 5 in the SI also show the temporal dynamics in the virus concentration per airway generation in the respiratory tract with the changes in airflow rate $Q_0$, immune response *r* and the virus reproduction rate *p* and highlights that rapid immune response can effectively control the spread of the virus downward the respiratory tract. Moreover, Fig. 6 in the SI also provides more information about the virus dynamics (change in virus concentration with ($C_0, Q$) and ($C_0, r$)). For instance, as shown in Fig. 7b, considering the increase in the immune response by T-cells *r* and decrease in the virus reproduction rate *p*, it greatly contributes to curb the virus load more effectively since the time taken to reduce the virus load decreases with higher *r* value. Interestingly, the airflow rate $Q_0$ has no major impact on viral dynamics (see Fig. 6 in SI). As observed from these graphs, it is clear that regardless of the changes in initial airflow rate $Q_0$, the virus load increases in the respiratory tract and a stronger immune response can effectively can control the spread of virus over the respiratory tract.

**Figure 8:**
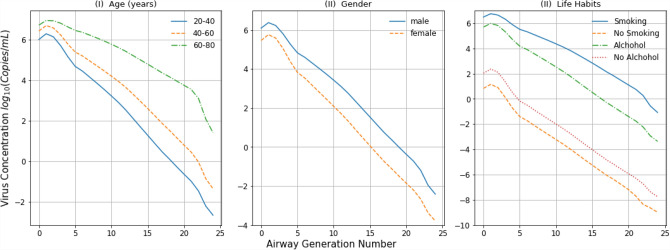
Change in virus concentration along the respiratory tract associated with changing age (*I*), gender (*II*), and lifestyle habits (*III*) when the exposure level $C_0$ is fixed to $10^7$ Copies/ml and the virus concentration is the average value computed for 21 days.

### Viral load with age, gender, and lifestyle

A major strength of our model is the ability to identify and understand risk factors that may contribute to poor outcomes.

For example, by incorporating real world data, Fig. 8 shows how age, gender and different lifestyle habits can potentially impact on the virus concentration along the respiratory tract because of the way these factors influence the immune response^22^. Figure 8(*I*) depicts the change in virus concentration across the respiratory tract with respect to three different age groups, whilst Fig. 8(*II*) shows the impact of gender. The model predicts that virus concentration increases on average as age increases, while women will have, on average, a lower virus concentration than men, because there is a gender difference in the average immune response to respiratory infections^22^. This does not mean that women are more likely to have a better outcome when infected as other factors can influence that too, but it does point to a potential area of concern for men. The model also predicts that the virus concentration increases in smokers and those that consume excessive amounts of alcohol, because of the impact these lifestyle habits can have on the immune response (Fig. 8*III*). This model also indicates that smoking may lead to a higher virus concentration than in those that consume alcohol excessively.

## Discussion

In response to the global COVID-19 pandemic, there has been considerable research undertaken to understand the virus, screen current and novel compounds to identify drugs that might be effective against SARS-CoV-2 and to develop vaccines (e.g., the study^26^ identified multiple drugs that were effective against SARS-CoV-2 and are now entering in vivo trials). This study reports the development of a model that maps the temporal virus concentration dynamics throughout the respiratory tract with respect to a range of parameters that affect the propagation of the virus and then explores how the model can provide deep insights into the temporal dynamics of an infectious virus along the respiratory tract. This model provides the opportunity to predict in silico, the ability of a respiratory virus to reach the lower parts of the lung and potentially cause disease, thus opening up opportunities to understand areas such as virus variants, virus-vaccine dynamics and antiviral drug efficacy and potential real world impact, without the requirement for in vivo data.

Data presented in this model highlights that virus concentration changes over time and multiple factors influence not only the concentration but also the location of the virus. Initially, virus will replicate in the upper respiratory tract and then progress into the lower parts, potentially to areas where tissue damage can lead to clinical symptoms and disease. This model allows an understanding of the distribution of the virus over time, while incorporating other factors such as immunocompetence and lifestyle habits. If designing antiviral drugs it is essential to understand the temporal distribution of virus in the respiratory tract—the ’where is it and when’ question? There is potential to focus on drugs that target specific areas of the respiratory tract at different stages of the infection, thus optimising the performance of the drug. For example, mucus adhesive nanoparticles that deliver a drug may be most effective for the early stages of any infection, but this model would predict that the same drug would not be majorly impactful on an older person after 5–7 days of an infection (when symptoms are appearing), because the model (Figs. 6 and  8) suggests that at that stage, on average, virus will have moved deeper into the lung and that is where the targeting is necessary. In contrast, for someone with a robust immune response, when symptoms appear around 5–7 days, this model predicts that the majority of virus is still in the upper respiratory tract, so rather than targeting the deep lung, a drug that would target the upper respiratory to block infection and onward transmission would be most effective.

Based on the mathematical models of airflow dynamics using the Navier Stokes equations, which are composed of partial differential equations used to describe motion of fluid (or air), the probability of the virus propagation and deposition deep into the lungs is high due to their micrometer scale diameter. However, our study has found a number of factors that affects the propagation dynamics of the virus that fluctuate as the virus flows through the branch generations of the airways. It is well known that the initial viral infection occurs in the upper respiratory tract and then spreads further down into the lung^26^. The main reason behind this is because the virus binds to ACE2 expressing cells. Figure 2b shows the deposition rate is much higher in the upper airways because of both impaction and sedimentation. This is due mainly to the propagation of virus under the advection mechanism, which encounters turbulence in the airflow leading to increased collision of viral particles on the airways walls. This leads to rapid replication in the upper respiratory tract early in the infection with some of this virus exiting through the nose and mouth, while a percentage will move down deeper into the respiratory system. There is also resistance against the flow of the viral particles due to the cilia, hair and mucus lining along the upper respiratory tract; in our analytical model, this resistance was accounted as the drag coefficient and slip correction factor in the computation of settling velocity $u_g$ in Eq. (34). Figure 2a shows the decrease in the viral particle velocity due to these obstructions and this also results in faster deposition rate that is dominated by the diffusion process deep down the lungs. As with most virus proliferation processes, Fig. 4c shows that the replication process can lead to a 100–1000 fold increase in virus particle number during the initial 24–48 h of an infection. This replication rate is compromised as the immune system starts to attack the virus^27^. In our study, the virus replication was derived by numerically solving the systems of differential Eqs. (7)–(10) with clinical viral infection data of the virus infectious dose, immune response, and concentration of ACE2-expressed cells^3,21,26^. Other factors may influence the virus such as inhibitory factors in mucus or co-factors on cell surfaces that enhance infections rates or change cell type preferences. Affinity to ACE2 may also be an important factor. Once inside cells, virus dynamics may be also influenced by the innate metabolism of the infected cell or the innate immune response of the cell. As biological data evolves, these details can be incorporated into a multi-modal model allowing subtle tweaks that would enhance the individualization capabilities of the model^28–31^.

It is clear from this model that ACE2 expression is a key factor that determines virus infection, replication and distribution in the respiratory system. In fact, from our models, ACE2 expression distribution along with the immune response are key factors in determining infection outcome. It is known that a number of factors such as smoking, age, gender and race^2,32–34^ can lead to varying densities of ACE2-expressing cells and immune response levels, and these differences will have an impact on clinical outcome as well as how easily a person will transmit the virus to others. Our analytical model considers temporal dynamics in the density of ACE2-expressed cells along the branches of the airway tract, where there is greater ACE2-expressed cell density in the nose and mouth (airway generation 1–2) as well as in the alveoli region (airway generation 21–24). This complies with a recent study^12^ that suggested the oral mucosa has good expression of ACE2, while the study^35^ highlighted that the ACE2-expression seems predominantly greater in the AT2 (type II alveolar) cells that express high levels of ACE2 in the lung, while the upper airways have a broad distribution of ACE2. This is why the virus load is considerably higher in the top and lower sections of the respiratory track compared to branches in the middle airway generations, and this is shown in in Fig. 5a for the lower infected cell values. In addition, Fig. 5b also highlights the greater possibility of viral infection being distributed over the upper airways and also alveoli region due to the higher density of ACE2 cells. Despite the change in ACE2 expression levels (also the viral exposure level) Fig. 6 provides insights that a robust early immune response can effectively curb virus proliferation and limit spread to lower regions of the respiratory tract. This has important implications for the vaccine rollout across the world. Secondly, while ACE2 distribution is clearly important, the ability of the virus to bind to ACE2 is also vital. As variants emerge, changes in the Spike protein are impacting on the ability for Spike to bind to ACE2. Already changes such as N501Y, have been shown to increase the viruses ability to bind to ACE2^36^.

It is believed that close contact with an infected person increases the risk of receiving a large dose of virus. However, it is still unclear what impact the concentration of the initial exposure dose has on clinical outcome. It is important to also note that symptoms do not necessarily correlate with virus load by nasal swab as asymptomatic people often have similar RT-PCR Ct values to symptomatic people, indicating that asymptomatics can potentially transmit virus as efficiently as symptomatics. Outcome of infection may be influenced strongly by initial exposure load however because if the initial dose is high, this may overwhelm the initial front line defence and move rapidly to the lung where serious disease is caused. This can be understood further analyzing the model outcomes depicted in Fig. 6. Independent of the initial dose $C_0$, the virus concentration increases but will then decrease or continue to increase, depending on the immune response that is initiated as shown in Fig. 6. As shown in Fig. 6b, when the immune response is strong enough, the peak virus persistence duration is reduced and hence the chance of advancing viral infection is very minimal. On the other hand, if the immune response is sub-optimal as in 6b(*I* and *II*), then there is opportunity for the virus to overcome it. This indicates clearly that the likelihood of an infection overcoming the initial immune response and moving deeper into the respiratory tract is dependent, not only on the initial viral load but also on the robustness of the immune response, as our model shows (Figs. 6 and 7 in SI).

It is known that there are particular cohorts of people that are most at risk of suffering from severe COVID-19 and death. Age is clearly the major predictor of likely outcome with the likelihood of severe disease or death increasing with age, but other factors such as obesity, diabetes and smoking can also contribute to an increased risk of a poor outcome^37,38^. Some studies have suggested that the replication rate of the virus may change depending on the cell type it has infected. For instance, smoking and alcohol consumption affect certain immune cell compositions in the innate immune response (e.g., natural killer cells, macrophages) as well as in the adaptive immune response (e.x., T- helper cells, T regulatory cells). This in turn facilitates the progression of virus and increase the virus load along the respiratory tract, as a result^39^. Also, the T-cell composition vary with sex depending on a number of factors such as environmental factors, nutrition status and the composition of the microbiome^40^. Moreover, differences in airway dimensions as well as the inlet air volume between adult and children^23^ could influence on the spread of viral infection dynamics. However, due to lack of airway dimensions with respect to age, we assumed the airway dimensions given in Table 1 in SI as the average airway dimensions over age and considered only the impact of immune response. All in all, these factors allow virus to progress and then spread along the respiratory tract. That is why aged people are more susceptible to the viral infection than young whilst male are more vulnerable to the virus than female. Although the data is not clear enough to include in the model at this stage, however, as more information becomes available the model can be rapidly adapted to take this into account. The simulation reported in Fig. 8 helps to explain how different lifestyle habits contribute to this poorer outcome. This implies that the computational model can serve as a prediction tool for people based on parameters such as lifestyle habits and a score could be allocated to this risk, allowing classification of people, thus helping people to understand their own personal risk.

## Conclusion

Biological understanding of SARS-CoV-2 is vital; how it infects, how it causes disease etc. Alongside that however is the vital need to build a wall of knowledge that will help to limit the impact of it and other pandemic and endemic viruses in the future. Accurate, rapid in silico models that incorporate real world data and evolve with that data will allow rapid identification of threats, rapid identification of vulnerable people to disease and rapid identification of drugs that are targeted correctly and effectively. The model proposed here lays the foundations of this and creates a framework that can be evolved and iteratively improved. It is a model that can predict outcomes before the biology confirms them and can be refined to improve the predictability and the quantification of risk associated with known and unknown threats going forward.

## Supplementary Information

Supplementary Information.
