# Supplementary material for: Virus particle propagation and infectivity along the respiratory tract and a case study for SARS-CoV-2
Source: Sci Rep. 2022 May 10;12:7666. doi: 10.1038/s41598-022-11816-2 (PMC9088735; doi:10.1038/s41598-022-11816-2)
Supplement: Supplementary file 1 — Supplementary Information. [file 41598_2022_11816_MOESM1_ESM.pdf]

Virus Particle Propagation and Infectivity along the Respiratory Tract and a Case Study for SARS-CoV-2(Supplementary Material)

Impedance (Z) airflow rate (Q) and airflow velocity (u)

Based on the conservation of fluid flow, the following conditions hold at a bifurcation junction as represented in Fig. 1.

$$Q_{(L_0,out)} = Q_{(L_{l1},in)} + Q_{(L_{r1},in)} \quad \text{and } P_{(L_0,out)} = P_{(L_{l1},in)} = P_{(L_{r1},in)}, \tag{1}$$

where Q- flow rate, P- pressure, L_{l1} and L_{r1} are respectively the length of the left (l) and right (r) branches of the parent airway of length L_0 (or in generation 1, G_1). The subscript *in* and *out* stands for the inlet and outlet of the airway, respectively.

Based on the circuit theory (i.e., Ohm's law; $V = IR$, where V-voltage, R-resistance and I-current), the impedance (Z), resistance against the airflow, is then computed as $Z = P/Q$[1]. Thus, the following relationship can be derived to compute the impedance at the junction (i.e., outlet of the pipe) as

$$\frac{1}{Z_{(L_0,out)}} = \frac{1}{Z_{(L_{l1},in)}} + \frac{1}{Z_{(L_{r1},in)}}. \tag{2}$$

Thereby, the impedance at the inlet of the parent airway is computed as

$$Z_{(L_0,in)} = Z_{L_0} + Z_{(L_0,out)}, \tag{3}$$

where Z_{L_0} is the impedance of the parent airway (i.e., G_0) and computed based on the following procedure.

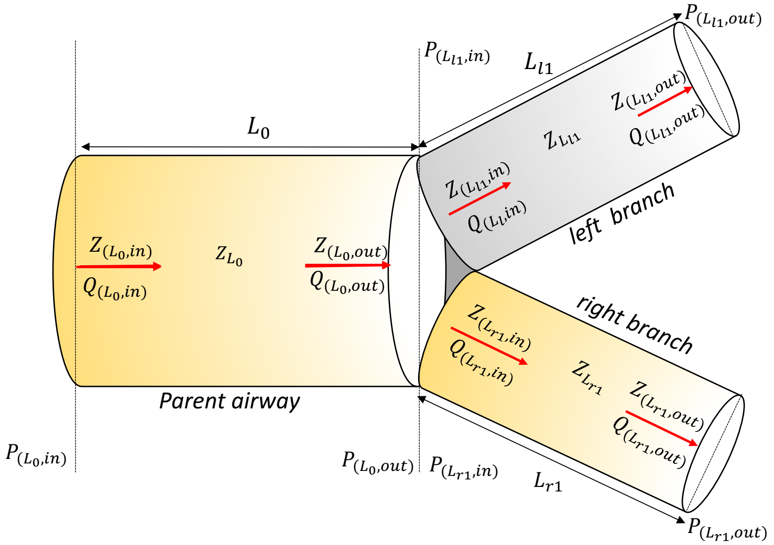

Figure 1. Bifurcation of the first airway generation (G_0), i.e., trachea into bronchus(this image was created by using Microsoft PowerPoint version 16.57 and can be accessed here).

Following the definition of impedance Z, i.e., $Z = P/Q$, and the concepts in fluid dynamics, the impedance of the parent airway Z_{L_0} is expressed as

$$Z_{L_0} = \frac{8\mu L_0}{\pi r_{L_0}^4}, \tag{4}$$

where μ- airflow viscosity and r_{L_0} is the airway diameter. By plugging Eqn. 4 in Eqn. 3, the impedance at the inlet of the parent airway can be calculated as

$$Z_{(L_0,in)} = \frac{8\mu L_0}{\pi r_{L_0}^4} + Z_{(L_0,out)}. \tag{5}$$

Now, at the bifurcation junction, the inlet flow rates into the left and right branches of the parent airway as well as its outlet pressure are then computed as

$$Q_{(L_{l1},in)} = Q_{(L_0,out)} \frac{Z_{(L_{r1},in)}}{Z_{(L_{l1},in)} + Z_{(L_{r1},in)}}, \tag{6}$$

$$Q_{(L_{r1},in)} = Q_{(L_0,out)} \frac{Z_{(L_{l1},in)}}{Z_{(L_{l1},in)} + Z_{(L_{r1},in)}}, \tag{7}$$

$$P_{(L_0,out)} = Q_{(L_0,out)} Z_{(L_0,out)} \tag{8}$$

$$Q_{(L_{l1},in)} = Q_{(L_{l1},out)} \quad \text{and} \quad Q_{(L_{r1},in)} = Q_{(L_{r1},out)} \tag{9}$$

As Fig. 1 represents the bifurcation (branching) of the respiratory tract as airway generations, a backward process is followed to compute the airflow rates, and then the velocity profiles over the respiratory tract.

Having computed the impedance of each airway generation, the airflow rate Q is then computed by using Eqns. 6-9. Finally, the $Q = uA$ relationship is used to compute the airflow velocity profile of each airway generation.

Virus Concentration Model Derivation ($C_{G_i}(x,t)$)

To derive a model for characterizing the virus propagation along a respiratory airway generation (G_i), consider a control volume V of depth δx with a cross sectional area A as illustrated in Fig. 1b in the main document (i.e., $V = A\delta x$). As Fig. 1b illustrates, when virus particles enter to an airway (shown as gray circles), they deposit on the airways walls and subsequently the virus infection takes place. As a consequence of the virus infection, more virus particles are reproduced (blue circles). The number of virus particles in the control volume (i.e., virus concentration, $C_{G_i}(x,t)$) can be expressed as $V\frac{dC_{G_i}(x,t)}{dt}$. Then, the accumulation of the virus in the control volume V can be formulated by considering the mass balance, i.e., virus particle in-flux (f_{in}) and interior flux-loss to the airway surface (f_{loss}) and out-flux (f_{out}) of virus particles as follows:

$$\begin{aligned}
V\frac{dC_{G_i}(x,t)}{dt} &= f_{in} + f_{reproduced} - (f_{out} + f_{loss}), \\
V\frac{dC_{G_i}(x,t)}{dt} &= Q_{in}A + pVC_{G_i} - (Q_{out}A + kVC_{G_i}), \\
\frac{dC_{G_i}(x,t)}{dt} &= -\frac{Q(x+\delta x,t) - Q(x,t)}{\delta x} - (k-p)C_{G_i}(x,t), \\
\frac{dC_{G_i}(x,t)}{dt} &= -\frac{dQ(x,t)}{dx} + (p-k)C_{G_i}(x,t),
\end{aligned} \tag{10}$$

where $k(s^{-1})$ and $p(s^{-1})$ are respectively the virus deposition rate on the airway surface and virus reproduction rate and Q is the airflow rate through the control volume $Q_{in} = Q(x,t)$ and $Q_{out} = Q(x+\delta t)$. Since the virus particles flow under the advection and diffusion mechanisms, the airflow rate Q can be formulated as the total airflow due to advection and diffusion, and hence,

$$Q(x,t) = u_i C_{G_i}(x,t) - D\nabla C_{G_i}(x,t), \tag{11}$$

where u_i is the air flow velocity at the G_i^{th} airway generation and D is the diffusion coefficient. Hence, the rate of change of air flow rate along the x-axis (in practice, $x-$axis implies the direction to which air flows), $\frac{dQ(x,t)}{dx}$, can then be written as

$$\frac{dQ(x,t)}{dx} = \frac{\partial C_{G_i} u_i}{\partial x} - D\frac{\partial C_{G_i}^2}{\partial x^2}. \tag{12}$$

When the airflow achieves its settling velocity (i.e., when the airflow flow reaches its steady state), $\nabla u_i = \frac{\partial u_i}{\partial x} = 0$ and D is a constant. So, Eqn. 12 can be simplified into the following expression:

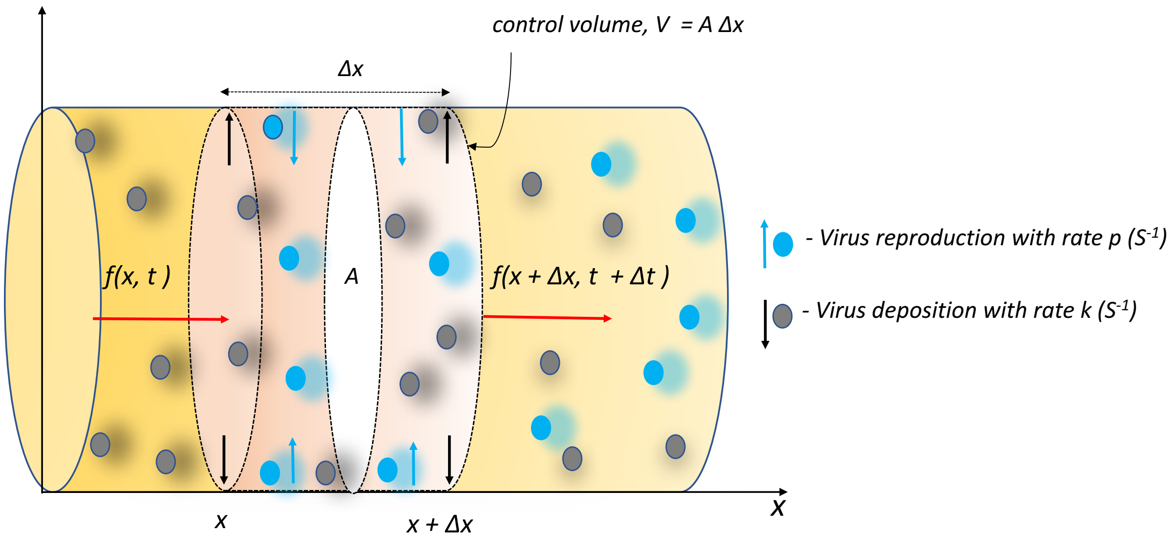

Figure 2. Virus particle propagation through a control volume of an airway generation with deposition rate k and reproduction rate p(this image was created by using Microsoft PowerPoint version 16.57 and can be accessed here).

$$\frac{dQ(x,t)}{dx} = u_i \frac{\partial C_{G_i}}{\partial x} - D \frac{\partial C^2_{G_i}}{\partial x^2}. \tag{13}$$

Based on the mass balance of the fluid flow without any reproduction and absorption of virus in Eqn. 10, the following relationship in Eqn. 14 holds regardless if the flow is due to advection or diffusion, and is represented as[2].

$$\frac{dC_{G_i}(x,t)}{dt} = -\frac{Q(x,t)}{dx}. \tag{14}$$

By plugging Eqns. 13 and 14 in Eqn. 10), the change in virus concentration along an airway can be represented in Eqn. 15. This is normally called the governing equation that represents the dynamics in virus concentration along the respiratory tract, and is represented as

$$\frac{\partial C_{G_i}(x,t)}{\partial t} + u_i \frac{\partial C_{G_i}(x,t)}{\partial x} - D \frac{\partial C_{G_i}(x,t)^2}{\partial x^2} + (p-k) C_{G_i}(x,t) = 0, \tag{15}$$

for $i = 0, 1, \cdots, 23$. Suppose the initial inlet virus concentration is C_{G_0}, i.e., $C_{G_0}(0,0) = C_{G_0}$, then the solution for Eqn. 15 can be derived by applying the following transformation:

$$C_{G_i}(x,t) = g(x,t) e^{(\alpha x - \beta t)} \quad \text{with} \quad \alpha = u_i/2D \quad \text{and} \quad \beta = K + u_i^2/4D, \tag{16}$$

where $K = k - p$. Then the partial derivatives of Eqn. 16 with respect to time t and x are

$$\frac{\partial C_{G_i}}{\partial t} = e^{(\alpha x - \beta t)} \frac{\partial g}{\partial t} - \beta g(x,y) e^{(\alpha x - \beta t)}, \tag{17}$$

$$\frac{\partial C_{G_i}}{\partial x} = \alpha g(x,t) e^{(\alpha x - \beta t)} + e^{(\alpha x - \beta t)} \frac{\partial g}{\partial x}, \tag{18}$$

$$\frac{\partial^2 C_{G_i}}{\partial x^2} = \alpha^2 g(x,t) e^{(\alpha x - \beta t)} + 2\alpha e^{(\alpha x - \beta t)} \frac{\partial g}{\partial x} + e^{(\alpha x - \beta t)} \frac{\partial^2 g}{\partial x^2}. \tag{19}$$

By plugging these derivatives in Eqn. 15, it can be converted in the form of the standard steady state diffusion equation in terms of $g(x,t)$ as given in Eqn. 20:

$$\frac{\partial g}{\partial t} = D\frac{\partial^2 g}{\partial x^2}. \tag{20}$$

The general analytical solution of Eqn. 20, the virus concentration, $C_{G_i}(x,t)$, at a point x and time t can be written as

$$g(x,t) = \frac{C_{G_{i-1}}}{2\sqrt{4\pi Dt}} e^{\left[-\frac{x^2}{4Dt}\right]}, \tag{21}$$

where $C_{G_{i-1}} = 2C_{G_0}$ only at $t = 0$. This is because the $C_{G_{i-1}}$ divides equally into two when virus enters into the two branching airways of the G_i generation.

The solution of Eqn. 15 can then be expressed as

$$C_{G_i}(x,t) = \frac{C_{G_{i-1}}}{2\sqrt{4\pi Dt}} e^{\left(\frac{-x^2}{4Dt}\right)} e^{(\alpha x - \beta t)}. \tag{22}$$

By substituting the values of α and β given in Eqn. 16 in Eqn. 22 and then re-arranging the terms in the exponential function, the final solution of Eqn. 15 can be expressed as follows

$$C_{G_i}(x,t) = \frac{C_{G_{i-1}}}{\sqrt{4\pi Dt}} e^{\left(-\frac{1}{4}\frac{(x-u_i t)^2}{Dt} - (k-p)t\right)}. \tag{23}$$

This expression presents the change in the virus concentration along an airway over time along with the effects of virus reproduction and deposition.

At the steady state, $\frac{\partial C_{G_i}}{\partial t} = 0$ and u is also a constant. Then, the Eqn. 15 can be simplified as

$$\begin{aligned} u_i\frac{\partial C_{G_i}}{\partial x} - D\frac{\partial^2 C_{G_i}}{\partial x^2} &= -(k-p)C_{G_i}, \\ \frac{\partial}{\partial x}(u_i C_{G_i} - D\frac{\partial C_{G_i}}{\partial x}) &= -(k-p)C_{G_i}. \end{aligned} \tag{24}$$

Note that according to Eqn. 11, $uC_{G_i} - D\frac{\partial C_{G_i}}{\partial x} = Q(x,t)$. The analytical solution of Eqn. 24 can be derived by transforming it into the following quadratic equation:

$$D\phi^2 - u_i\phi - (k-p) = 0, \tag{25}$$

where $\phi = \frac{\partial C_{G_i}(x,t)}{\partial x}$. The solution of Eqn. 25 can be written as $\phi = \frac{u_i \pm \sqrt{u_i^2 + 4D(k-p)}}{2D}$. Since the concentration cannot be negative or an imaginary number, the condition $u_i^2 + 4D(k-p) > 0$ should hold, that is $u_i > 2\sqrt{D(p-k)}$. Hence, the final solution of Eqn. 25 is

$$C_{G_i}(x) = Ae^{(\phi x)} \tag{26}$$

where A is an integration coefficient that has to be determined. To compute the value of A in the steady state considering Eqn. 24, this can be represented as

$$\frac{d}{dx}(u_i C_{G_i} - D\frac{dC_{G_i}}{dx}) = -(k-p)C_{G_i}. \tag{27}$$

It is given in Eqn. 12 that $Q = uC - D\frac{dC_{G_i}}{dx}$. At $x = 0$, from 26, $C_{G_i}(x=0)A$ and $\frac{dC_{G_i}(x)}{dx}|_{x=0} = A\phi$ and $Q(x=0) = u_i C_{G_i}(x = 0) = u\frac{C_{G_{i-1}}}{2}$. Hence, according to 27,

$$\begin{aligned} u\frac{C_{G_{i-1}}}{2} &= u_iC(x=0) - D\frac{dC_{G_i}(x)}{dx}|_{x=0} = A\phi, \quad (28)\\ (u_i-\phi D)A &= u_i\frac{C_{G_{i-1}}}{2},\\ A &= \frac{u_iC_{G_{i-1}}}{2(u_i-\phi D)}. \end{aligned}$$

Now, A can have two values A_1 and A_2 as follows

$$A_1 = \frac{C_{G_{i-1}}u_i}{u_i - \sqrt{u_i^2+4D(k-p)}} \rightarrow \text{when} \quad \phi = \frac{u_i+\sqrt{u^2+4D(k-p)}}{2D}, \quad (29)$$

$$A_2 = \frac{C_{G_{i-1}}u_i}{u_i + \sqrt{u_i^2+4D(k-p)}} \rightarrow \text{when} \quad \phi = \frac{u_i-\sqrt{u_i^2+4D(k-p)}}{2D}. \quad (30)$$

Considering Eqn. 29, $A_1 \geq 1$ as $u > u-\sqrt{u^2+4D(k-p)}$, and this turns out that according to Eqn. 26, $C_{G_i}(x)$ is increasing with x. On the other hand, according to Eqn. 30, $A_2 \leq 1$ as $u_i < u_i+\sqrt{u_i^2+4D(k-p)}$, hence $C(x)$ decreases with x. In practice, the virus concentration should decrease when the virus travels along the airway tract. Thus, A_2 is the most suitable value for A.

Finally, by substituting the A and ϕ values derived from Eqn. 28 and Eqn. 25 in eqn. 26, the steady state virus concentration along the airway tract, $C_{G_i}(x)$, can be written as

$$C_{G_i}(x) = \frac{C_{G_{i-1}}u_i}{u_i+\sqrt{u_i^2+4D(k-p)}} exp\left[\left(\frac{u_i-\sqrt{u_i^2+4D(k-p)}}{2D}\right)x\right], \quad (31)$$

for $u > 2\sqrt{D(p-k)}$ and $i = 0,1,\cdots,23$.

Settling Velocity (u_g)

The maximum velocity that a virus particle can achieve is known as its settling velocity; velocity of a particle when the total external forces acting on it becomes zero. Fig. 3, for instance, depicts a virus particle of mass m, diameter d_p and density ρ_p that enters to an an airflow of density ρ_f at $t = 0$ with velocity u_0 and then propagates along an airway with velocity u_f under three external forces given in Eqns. 32- 33. We assume the virus particle achieves its settling velocity u_g at $t = T$ after traveling L_T distance. When the virus particle moves through a projected area A_p at an arbitrary time t, its relative velocity (velocity with respect to the airflow) can be expressed as $u_{p,t} = u_f - u_p$, and it also gains an acceleration due to the imbalance of the external forces that act on it, and this includes

$$\begin{aligned} F_e &= mg \quad \text{(gravitational force)}, \quad (32)\\ F_D &= \frac{C_Du_{p,t}^2\rho_fA_p}{2C_c} \quad \text{(drag force)},\\ F_b &= \text{mass of fluid displaced} \times \text{acceleration form the external force},\\ &= [\frac{m}{\rho_p}\rho_f]\times g \quad \text{(Buoyant force)}, \quad (33) \end{aligned}$$

where C_D is the drag coefficient and C_c is the slipping coefficient. The Newton's second law (i.e., $F = \frac{d(mu_p)}{dt}$) can then be used to express relationship of these three forces as

$$\begin{aligned} F_e - F_D - F_b &= \frac{d(mu_p)}{dt},\\ \frac{d(u_p)}{dt} &= \frac{g(\rho_p-\rho_f)}{\rho_p} - \frac{C_Du_{(p,t)}^2\rho_fA_p}{2C_cm}. \quad (34) \end{aligned}$$

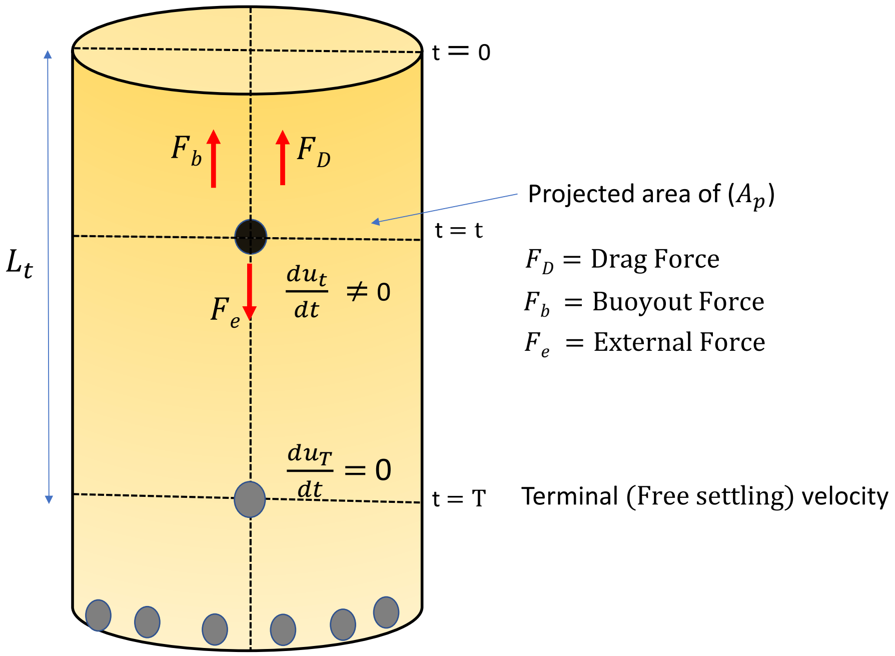

Figure 3. Virus particle propagation under gravity. The virus particles first achieve settling velocity and then deposition on the airway walls(this image was created by using Microsoft PowerPoint version 16.57 and can be accessed here).

According to the definition of the Reynolds's number, Reynolds's number of the virus particle is $Re_p = \frac{\rho_p d_p u_{(p,t)}}{\mu}$ and hence the relative velocity can be expressed as $u_{(p,t)} = \frac{Re_p \mu}{\rho_p d_p u_{(p,t)}}$. Moreover, assuming that the spherical shaped virus particles, the weight m and cross sectional area A_p of the virus particle can be expressed as $m = \frac{\pi d_p^3 \rho_p}{6}$ and $A_p = \frac{\pi d_p^2}{4}$, respectively. By plugging these terms in Eqn. 34, then Eqn. 35 expresses the change in particle velocity u_p as

$$\frac{du_p}{dt} = \frac{g(\rho_p - \rho_f)}{\rho_p} - \frac{3\mu C_D Re_p \rho_f}{4C_c d_p^2 \rho_p^2} u_{(p,t)}, \tag{35}$$

where g is the acceleration gravity and

$$\begin{aligned} Re_p &= \rho \frac{u_{p,t} d_p}{\mu} \quad \text{(Particle Reynolds number)}, \\ C_D &= \frac{24}{Re_p}(1 + 0.15 Re_p^{0.687}) \quad \text{(Particle Drag Coefficient)}, \\ C_c &= 1 + \frac{2\lambda}{d_p}(1.257 + 0.4 e^{-1.1 \frac{d_p}{2\lambda}}) \quad \text{(Cunningham slip correction factor)}. \end{aligned}$$

The virus particle achieves the settling velocity, u_g, when $\frac{du_p}{dt} = 0$ (i.e., Eqn. 35 = 0), hence the settling velocity can be expressed as

$$u_g = u_{(p,t)} = \frac{4C_c d_p^2 \rho_p}{3\mu C_D Re_p} \frac{(\rho_p - \rho_f)}{\rho_f} g. \tag{36}$$

Respiratory Airway Generations and Their Physiological Parameters

Additional Results

This section presents some aiming to give more in depth understanding about the viral dynamics along the respiratory tract.

Table 1. Physiological parameters of the respiratory airway generations taken from[3].

Generation number	G_0	G_1	G_2	G_3	G_4	G_5	G_6	G_7	G_8	G_9	G_{10}	G_{11}
	G_{12}	G_{13}	G_{14}	G_{15}	G_{16}	G_{17}	G_{18}	G_{19}	G_{20}	G_{21}	G_{22}	G_{23}
Length	12	4.76	1.9	.76	1.27	1.07	.9	.76	.64	.54	.46	.39
(L cm)	.33	.27	.23	.2	.165	.14	.12	.099	.083	.07	.059	.05
Diameter	1.8	1.22	.83	.56	.45	.35	.28	.23	.186	.154	.130	.109
(d_L mm)	.095	.082	.074	.061	.06	.054	.05	.047	.045	.043	.041	.04

Impact of Breathing and Virus size on Virus Deposition

In addition to the impact of the virus particle diameter d_p on the flow velocity u and deposition rate k presented in Fig. 3 in the main document, the inlet airflow rate Q_0 can also impact on the particle deposition. Fig. 4a$(I-IV)$ shows the impact of changes in the inlet airflow rate Q_0 on the airflow velocity while Fig. 4b$(I-IV)$ depicts the influence of the inlet airflow rate Q_0 on the virus deposition along the respiratory tract. The virus deposition rate increases with increasing inlet airflow rate and a higher deposition can be observed over the upper airway generations ($\sim G_0 - G_{15}$).

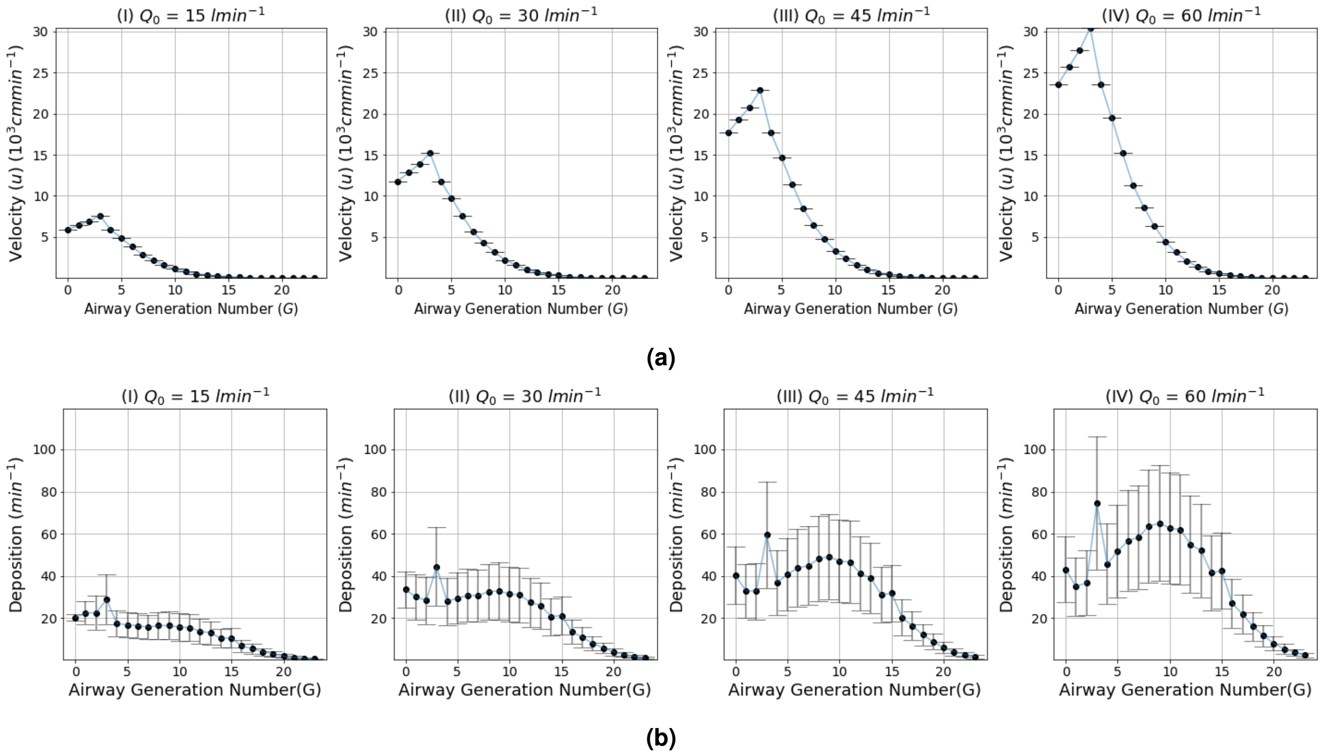

Figure 4. The impact of airflow rate Q on the airflow velocity V and particle deposition rate k; **(a)** airflow velocity V and **(b)** deposition rate k with respect to variations in the airflow rate Q the range 15-60 $lmin^{-1}$ (the diameter of the virus particle d_p changes in 70-130 μm).

Variability in Virus Load Over Airway Generations

Insights about the variability in the virus load over the respiratory tract is important particularly in early diagnosis and making timely treatments to control the virus spread and development into severe illness. This is critically important in treating patients with chronic diseases like diabetes. In this regard, Fig.5 depicts the variability in the virus load over the respiratory tract after 35 days given that a person exposed to the virus with an initial dose $10^5 Copies/ml$ and breathing rate is $30 lmin$. Since the ACE2 expressed cell density is high around the mouth as well as the alveolar region, the variability in the virus load is high.

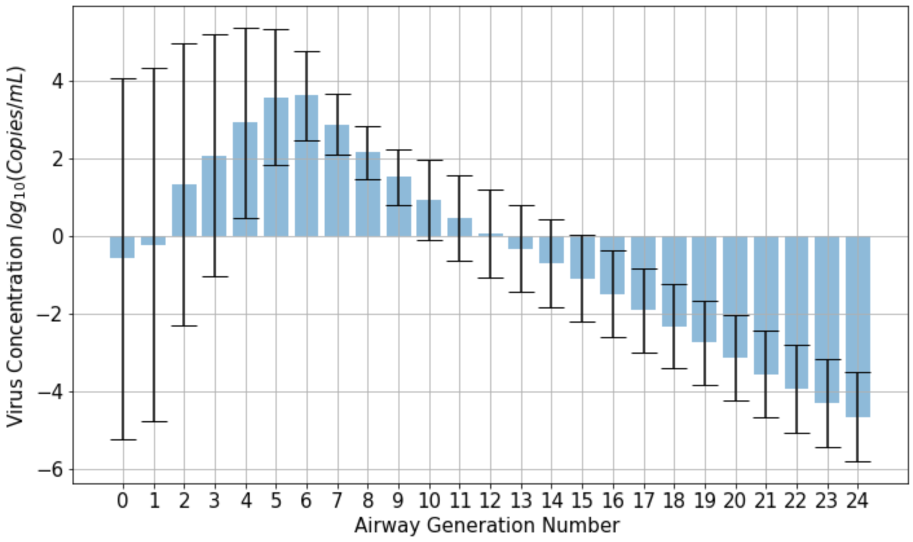

Figure 5. Average virus load in each of the airway generation over a period of 30 day. ACE2 concentration vary over the the upper ($\sim 1-3$ generations) and lower ($\sim G_{21}-G_{23}$ generations) regions as $\mathcal{N}(5-7,.71)$ and it is $\mathcal{N}(5.83,0.71)$ over the remaining airway generations, and air flow rate Q_0 is $30lmin^{-1}$ and virus diameter d_p is $70nm$.

Impact of Airflow Rate, Clearance of Infected Cells, T-cell Proliferation and Exposure Level on the Temporal Virus Concentration Dynamics

In order to get broader understanding about the variability in virus dynamics shown in Fig. 7 in the main document, this section presents change in virus concentration with respect to different parameters. Considering the immune response by T-cells in the infection model given by Eqns. 7-10 in the main document), Fig. 6 depicts the impact of the initial infectious dose C_0, airflow rate Q_0, and T-cell proliferation rate r on the variability in temporal viral dynamics. Fig. 6a$(I-III)$ show that, despite a change in the initial airflow rate Q_0, the virus concentration decreases along the respiratory tract with the increase in the immune response by T-cells. It can be expected that higher airflow rate can take a greater amount of virus downwards the respiratory tract. Whereas, considering Fig. 6(I) and (II), a higher virus concentration can be observed in the lower region of the respiratory tract with low airflow rate. Thus, interestingly, the airflow rate has no major impact on viral dynamics. Moreover, in Fig. 6b$(I-III)$, it can also be observed that there is a decrease in virus concentration along the respiratory tract with decreasing the exposure level and increase in the immune response by T-cells. Considering the increase in T-cell proliferation rate r, it greatly contributes to curb the virus lconcentration more effectively since the persistance of high virus concentration decreases with higher r value.

Impact of Effective Dose and Immune Response by T-cells

The amount of virus required to initiate an infection is still unclear due to a lack of clinical evidence. The computational model investigated in this study based on the model in Eqn. 5 suggests that the virus load along an airway increases with higher exposure levels. Nonetheless, the chance of initiating an infection with a small dose of virus will likely be small as the immune system can effectively fight against the virus based on Eqns. 7-10. However, in the event of high exposure to the virus, the immune response may not be strong enough to fight against the virus replication, and this increases the probability of the infection reaching the lungs. To examine this in greater detail, we simulated the impact of different infectious doses on the virus concentration across different immune response levels.

In addition to Fig.6 in the main document, to understand the impact of the immune response by T-cells on the viral dynamics more in depth, Fig. 7 represents the impact of initial exposure dose C_0 and T-cell concentration T_0 on the virus load over the respiratory airway generations. Fig. 7a$(I-III)$ exhibit the temporal dynamics in virus concentration over airway generations using three different infectious doses, representing a low, medium and high concentrations. Having fixed the T-cell proliferation rate r to 6, the variability in virus load over the airway generations is shown for three different exposure levels in Fig. 7b$(I-III)$ when T_0 increases from 3×10^3 to 4×10^7 $Copies/\mu l$. The virus load decreases with increasing T_0. Hence, a stronger immune response by T-cells can effectively act against the virus replication. Fig. 7c$(I-III)$ also show the impact of change in T-cell proliferation rate r when the T_0 value increases from 3×10^3 to 4×10^7 $Copies/\mu l$ and C_0 is fixed to 10^7 $(Copies/ml)$. Similar to what observed from Fig. 7b$(I-III)$, greater T_0 level helps in controlling the virus proliferation effectively. Thus, as observed

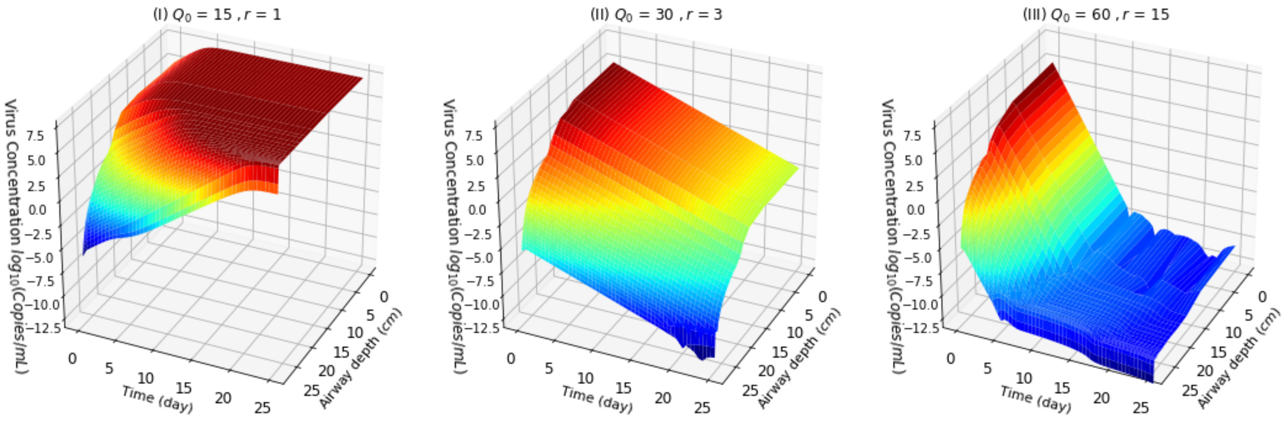

(a)

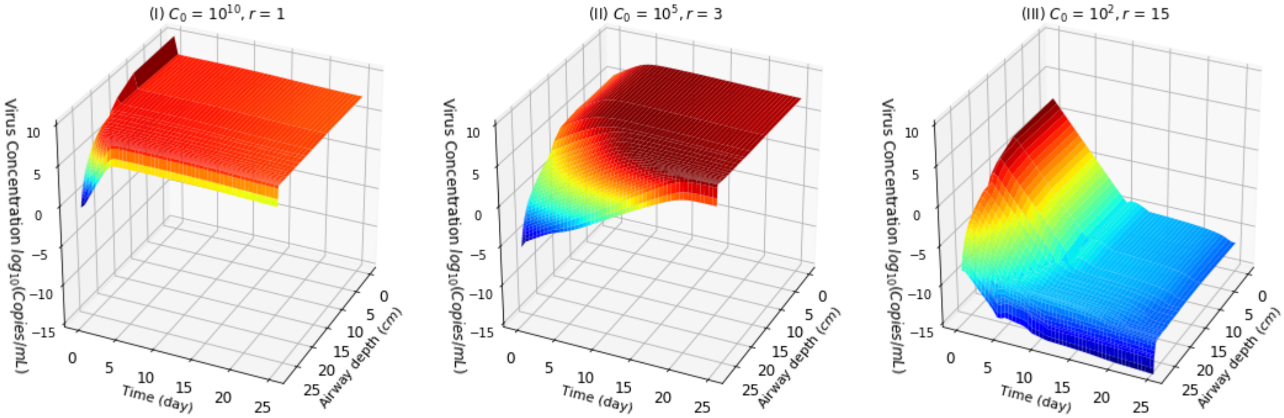

(b)

Figure 6. Variability in virus dynamics integrated with the immune response by T-cells over the respiratory airways with respect to the change in three parameters; **(a)** air flow rate Q_0 and T-cell proliferation rate r for a fixed exposure level $C_0 = 10^7 Copies/ml$ **(b)** T-cell proliferation rate r and exposure level C_0 for fixed air flow rate $Q_0 = 30 lmin^{-1}$; where virus particle diameter d_p varies over the range of 70-140 μm.

from these graphs, regardless of the exposure dose, when the $T_0 > 10^5$ $Copies/\mu l$ and T-cell proliferation rate $r > 10$ day^{-1} , the chance of susceptibility to the viral infection is minimal.

This model shows that there is a balance between both the infectious dose and the immune system of the person. If the initial starting amount of circulating T-cells is constant, the effectiveness of the response is critical and dictates whether the virus progresses to the lower parts of the lung or not. An effective immune response can potentially deal with a larger infectious dose, while a weaker immune response may struggle to manage even a small dose. Importantly, this result also suggests that there is a minimum dose, below which, disease is highly unlikely, and a dose, above which an immune response will struggle to cope with.

References

1. Chahibi, Y. *A molecular communication framework for modeling targeted drug delivery systems*. Ph.D. thesis, Georgia Institute of Technology (2016).
2. Jaiswal, D. K., Kumar, A. & Yadav, R. R. Analytical solution to the one-dimensional advection-diffusion equation with temporally dependent coefficients. *J. Water Resour. Prot.* **3**, 76–84, DOI: 10.4236/jwarp.2011.31009 (2011).
3. Balashazy, I., Hofman, W. & Maratonen, S. Inspiratory particle deposition in airway bifurcation models. *Aerosol Sci.* **22**, 15–30, DOI: 10.1101/2020.03.26.20044487 (1991).

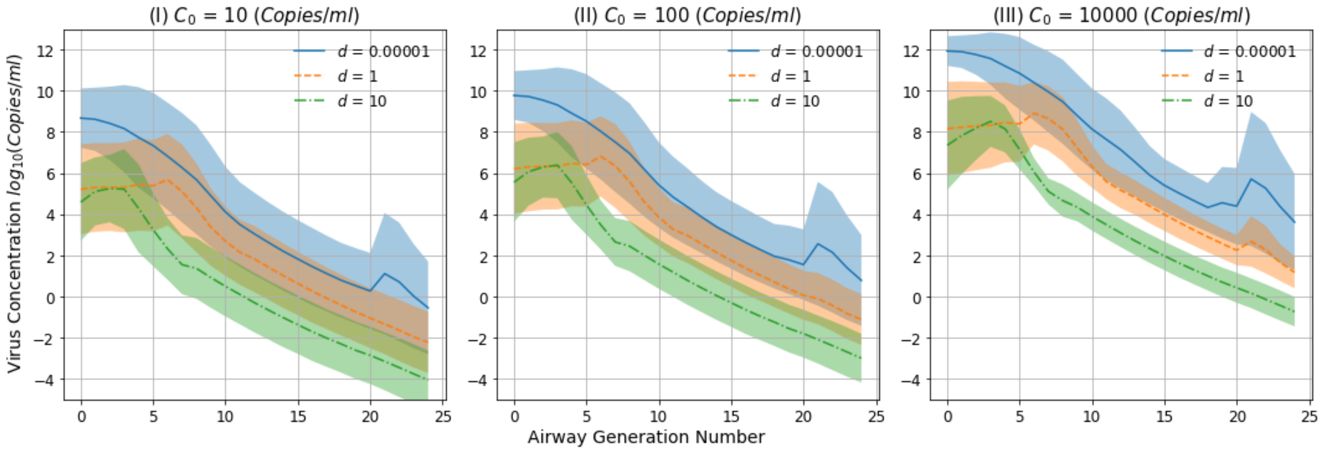

(a)

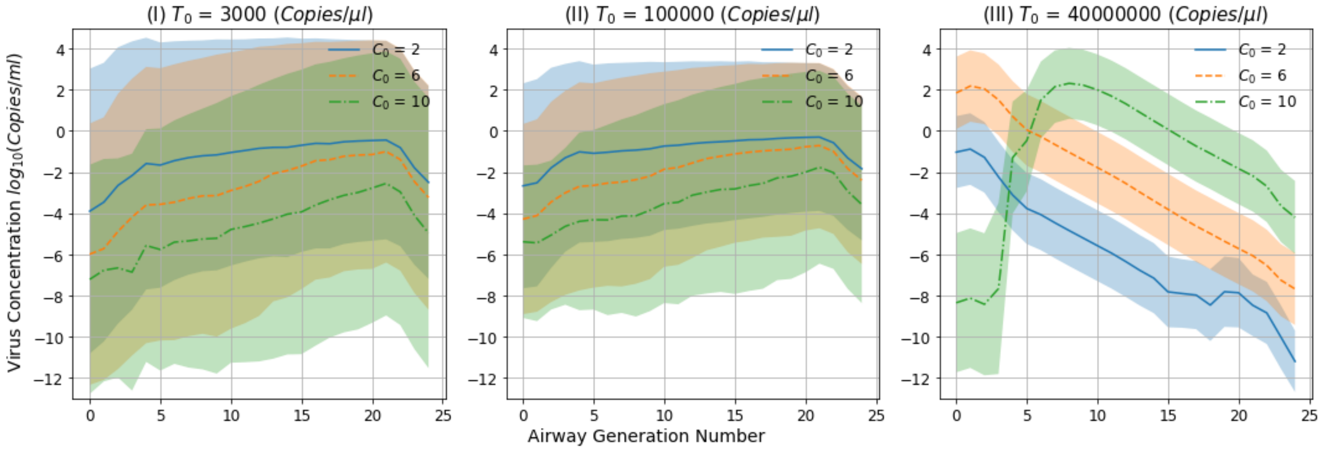

(b)

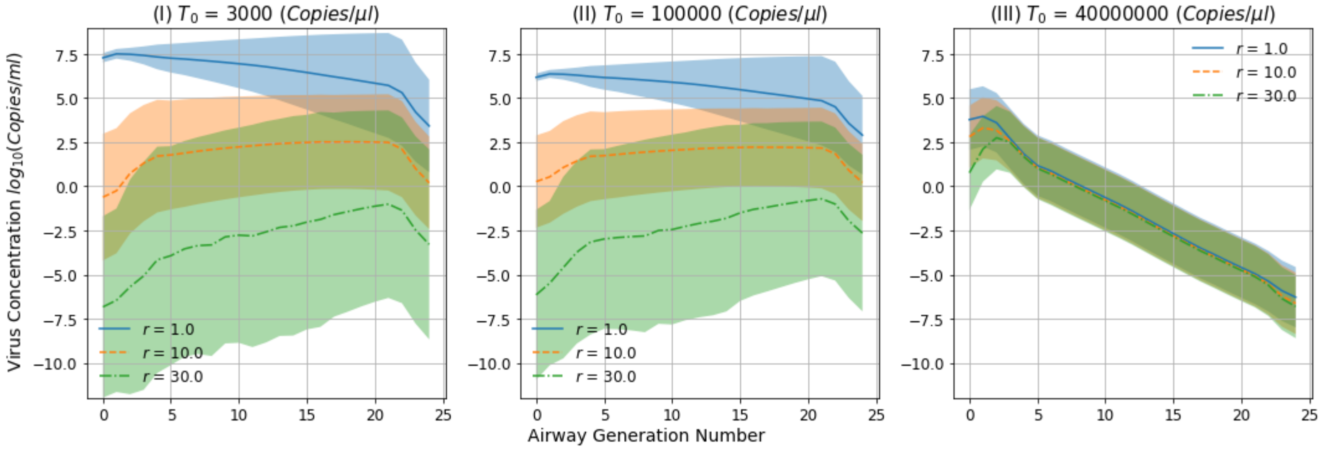

(c)

Figure 7. Total virus load dynamics with initial T-cell concentration T_0 and T-cell proliferation rate r;**(a)** three initial infectious doses (C_0) with $T_0 = 10^3\ Copies/\mu l$ and $r = 6\ day^{-1}$ **(b)** three exposure levels and T-cell levels with a fixed T-cell proliferation rate $r = 6\ day^{-1}$ and **(c)** three T-cell levels and T-cell proliferation rates with a fixed exposure level $C_0 = 10^7\ Copies/ml$; the time period considered here is for 30 days, the initial airflow rate $Q_0 = 30\ lmin^{-1}$, virus particle diameter $d_p = 70nm$.
